# Chronotype, Genetic Risk, Lifestyle, and Risk of Depression and Anxiety: A Prospective Cohort Study

**DOI:** 10.1002/mco2.70736

**Published:** 2026-04-08

**Authors:** Dongming Wang, Zhonghe Shao, Zhaomin Chen, Xingjie Hao, Wenzhen Li

**Affiliations:** ^1^ Department of Occupational & Environmental Health School of Public Health, Tongji Medical College, Huazhong University of Science and Technology Wuhan China; ^2^ Key Laboratory of Environment and Health and State Key Laboratory of Environmental Health (Incubating) School of Public Health Ministry of Education & Ministry of Environmental Protection, Tongji Medical College, Huazhong University of Science and Technology Wuhan China; ^3^ Department of Epidemiology and Biostatistics School of Public Health Tongji Medical College, Huazhong University of Science and Technology Wuhan China; ^4^ Jockey Club School of Public Health and Primary Care The Chinese University of Hong Kong Hong Kong China; ^5^ Shenzhen Research Institute of the Chinese University of Hong Kong Shenzhen China

**Keywords:** anxiety, chronotype, depression, genetic risk, lifestyle

## Abstract

We aimed to evaluate associations of chronotype, genetic risk, and lifestyle with depression and anxiety. A total of 242,391 participants without anxiety and depression at baseline in UK Biobank were included. During a total of 3,393,260.1 and 1,371,872.8 person‐years follow‐up, we found 11,824 (4.88%) incident depression and 10,051 (4.15%) incident anxiety cases, respectively. Compared with definite morning group, individuals with intermediate (HR = 1.09, 95% CI = 1.04‒1.13) and definite evening chronotype (HR = 1.45, 95% CI = 1.36‒1.55) have higher risks of depression, and individuals with definite evening chronotype (HR = 1.27, 95% CI = 1.18‒1.37) have a higher risk of anxiety. We found joint association between chronotype and genetic risk, those with high genetic risk and definite evening chronotype had the highest risk of depression (HR = 2.01, 95% CI = 1.81‒2.23) and anxiety (HR = 1.40, 95% CI = 1.24‒1.58). We also found joint association between chronotype and lifestyle, those with least healthy lifestyle and definite evening chronotype had the highest risk of depression (HR = 1.99, 95% CI = 1.65‒2.40) and anxiety (HR = 1.69, 95% CI = 1.36‒2.10). Individuals with evening chronotype are associated with higher risks of depression and anxiety.

## Graphical abstract

Compared with definite morning group, individuals with intermediate chronotype and definite evening chronotype have a higher risk of depression, and individuals with definite evening chronotype have a higher risk of anxiety. We also found joint association between chronotype and genetic risk/lifestyle with depression and anxiety. Individuals with evening chronotype are more likely to develop depression and anxiety.

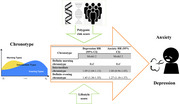

## 1 Introduction

Mental health disorders are considered as primary contributors to global disease burden [1], especially for depression and anxiety, no matter the age‐standardized prevalence or the age‐standardized disability‐adjusted life‐year (DALY) rate [2]. The burden of mental health is predicted to increase in coming decades in the worldwide, and untreated mental disorders are reported to be associated with a high suicide mortality [3] and economic burden [4]. Thus, exploring risk factors remains indispensable in mitigating the incidence of depression and anxiety.

Chronotype is the attitude of an individual to conduct activities with different times, which has been attracted more and more concern in recent years [5]. Chronotype was usually divided into definite morning, definite evening, and intermediate groups [6]. Definite morning was defined as those with earlier activity timing or sleep schedules, while definite evening was identified later activity peaks or delayed sleep timing. Studies have indicated that definite evening chronotype was related with several adverse health outcomes, including cardiovascular diseases [7], diabetes [6], obesity [8], etc. Some previous studies have assessed the relationship between chronotype and mental health [9, 10, 11]; however, the conclusion is debating and most studies focused on depression. Evidence regarding the relationship of chronotype with depression and anxiety needs to be evaluated comprehensively, especially for prospective cohort study.

Genetic risk has also been identified as a significant contributor to the occurrence of depression and anxiety [12, 13]. Individuals with high genetic risk using polygenic risk score (PRS) usually have higher risks of depression and anxiety. In addition, lifestyle was also associated with depression and anxiety. For instance, studies revealed that lifestyle intervention is efficiency for individuals with depression and anxiety [14, 15]. Assessing the association of chronotype and genetic risk/lifestyle with depression and anxiety could help us identify high‐risk populations in early stage. However, the role of genetic risk and lifestyle in the relationship of chronotype with depression and anxiety is unknown.

Thus, we conducted the present study from UK Biobank (UKB) to explore the relationship of chronotype with depression and anxiety. Meanwhile, we also explored the role of genetic risk and lifestyle in the above‐mentioned association.

## 2 Results

### 2.1 Baseline Characteristics and Incident Cases

The baseline characteristics of included participants are presented in Table 1. Among all the 242,391 participants, 130,401 (53.80%) were female and the mean age was 56.88 years. The numbers of individuals with definite morning chronotype, intermediate chronotype, and definite evening chronotype were 66,255, 156,206, and 19,930, respectively. Depression and anxiety individuals are more likely to be female, definite evening chronotype, least lifestyle, and high genetic risk. Table S1 presents the characteristics of included participants at baseline by chronotype.

**TABLE 1:** Descriptive characteristics of the participants and stratified by depression and anxiety.

Characteristics	Total (*n* = 242,391)	Depression (*n* = 11,824)	No depression (*n* = 230,567)	Anxiety (*n* = 10,051)	No anxiety (*n* = 232,340)
Age, mean ± SD (years)	56.88 ± 8.01	56.87 ± 8.07	56.88 ± 8.00	57.60 ± 8.00	56.85 ± 8.01
Gender					
Female	130,401 (53.80)	7522 (63.62)	122,879 (53.29)	6637 (66.03)	123,764 (53.27)
Male	111,990 (46.20)	4302 (36.38)	107,688 (46.71)	3414 (33.97)	108,576 (46.73)
Country					
England	231,756 (95.61)	11,595 (98.06)	220,161 (95.49)	9904 (98.54)	221,852 (95.49)
Wales	10,635 (4.39)	229 (1.94)	10,406 (4.51)	147 (1.46)	10,488 (4.51)
Education					
Higher	75,660 (31.21)	2790 (23.60)	72,870 (31.60)	2411 (23.99)	73,249 (31.53)
Upper secondary	83,222 (34.33)	4005 (33.87)	79,217 (34.36)	3367 (33.50)	79,855 (34.37)
Lower secondary	13,329 (5.50)	815 (6.89)	12,514 (5.43)	659 (6.56)	12,670 (5.45)
Vocational	29,647 (12.23)	1562 (13.21)	28,085 (12.18)	1297 (12.90)	28,350 (12.20)
No secondary education	39,249 (16.19)	2581 (21.83)	36,668 (15.90)	2255 (22.44)	36,994 (15.92)
Prefer not to answer	1284 (0.53)	71 (0.60)	1213 (0.53)	62 (0.62)	1222 (0.53)
Household income					
Less than £31,000	102,138 (42.14)	6576 (55.62)	95,562 (41.45)	5457 (54.29)	96,681 (41.61)
£31,000 and above	114,063 (47.06)	3838 (32.46)	110,225 (47.81)	3283 (32.66)	110,780 (47.68)
Missing data	26,190 (10.80)	1410 (11.92)	24,780 (10.75)	1311 (13.04)	24,879 (10.71)
Townsend deprivation index, mean ± SD	‒1.65 ± 2.88	‒0.93 ± 3.19	‒1.68 ± 2.85	‒1.15 ± 3.15	‒1.67 ± 2.86
Current employment status					
Employed	138,706 (57.22)	5502 (46.53)	133,204 (57.77)	4591 (45.68)	134,115 (57.72)
Retired	85,807 (35.40)	4361 (36.88)	81,446 (35.32)	4063 (40.42)	81,744 (35.18)
Unemployed, home	16,521 (6.82)	1865 (15.77)	14,656 (6.36)	1332 (13.25)	15,189 (6.54)
None of the above	935 (0.39)	60 (0.51)	875 (0.38)	43 (0.43)	892 (0.38)
Prefer not to answer	422 (0.17)	36 (0.30)	386 (0.17)	22 (0.22)	400 (0.17)
Chronotype					
Definite morning chronotype	66,255 (27.33)	2999 (25.36)	63,256 (27.43)	2733 (27.19)	63,522 (27.34)
Intermediate chronotype	156,206 (64.44)	7455 (63.05)	148,751 (64.52)	6251 (62.19)	149,955 (64.54)
Definite evening chronotype	19,930 (8.22)	1370 (11.59)	18,560 (8.05)	1067 (10.62)	18,863 (8.12)
Healthy lifestyle factor					
Smoking	23,165 (9.56)	1895 (16.03)	21,270 (9.23)	1384 (13.77)	21,781 (9.37)
Alcohol intake	52,310 (21.58)	2271 (19.21)	50,039 (21.70)	1965 (19.55)	50,345 (21.67)
Physical activity	113,682 (46.90)	6501 (54.98)	107,181 (46.49)	5321 (52.94)	108,361 (46.64)
TV viewing	71,436 (29.47)	4475 (37.85)	66,961 (29.04)	3736 (27.17)	67,700 (29.14)
Sleep time	60,262 (24.86)	3977 (33.63)	56,285 (24.41)	3192 (31.76)	57,070 (24.56)
Fruit and vegetable intake	38,900 (16.05)	2267 (19.17)	36,633 (15.89)	1814 (18.05)	37,086 (15.96)
Oily fish intake	102,104 (42.12)	5257 (44.46)	96,847 (42.00)	4331 (43.09)	97,773 (42.08)
Red meat intake	34,044 (14.04)	1661 (14.05)	32,383 (14.04)	1321 (13.14)	32,723 (14.08)
Processed meat intake	75,408 (31.11)	3729 (31.54)	71,679 (31.09)	3035 (30.20)	72,373 (31.15)
Lifestyle category					
Most healthy	139,401 (57.51)	5680 (48.04)	133,721 (58.00)	5137 (51.11)	134,264 (57.79)
Moderately healthy	96,450 (39.79)	5558 (47.01)	90,892 (39.42)	4497 (44.74)	91,953 (39.58)
Least healthy	6540 (2.70)	586 (4.96)	5954 (2.58)	417 (4.15)	6123 (2.64)
Genetic risk category					
Depression					
Low	81,178 (33.49)	3115 (26.34)	78,063 (33.86)	2746 (27.32)	78,432 (33.76)
Medium	80,730 (33.31)	3940 (33.32)	76,790 (33.30)	3383 (33.66)	77,347 (33.29)
High	80,483 (33.20)	4769 (40.33)	75,714 (32.84)	3922 (39.02)	76,561 (32.95)
Anxiety					
Low	80,797 (33.33)	3714 (31.42)	77,083 (33.43)	3166 (31.50)	77,631 (33.41)
Medium	80,797 (33.33)	3865 (32.69)	76,932 (33.37)	3335 (33.18)	77,462 (33.34)
High	80,797 (33.33)	4245 (35.90)	76,552 (33.20)	3550 (35.32)	77,247 (33.25)
BMI, mean ± SD	27.38 ± 4.66	28.62 ± 5.55	27.32 ± 4.61	27.98 ± 5.24	27.35 ± 4.64
Hypertension	66,332 (27.37)	4142 (35.03)	62,190 (26.97)	3448 (34.31)	62,884 (27.07)
Diabetes	11,724 (4.84)	946 (8.00)	10,778 (4.67)	629 (6.26)	11,095 (4.78)

*Note*: Continues variables are displayed as means ± SDs, and categorical variables are displayed as numbers (percentages).

Abbreviations: BMI, body mass index; SD, standard deviation.

### 2.2 Association Between Chronotype and Depression/Anxiety

Table 2 reveals the relationship of chronotype with incident depression and anxiety. During a total of 3,393,260.1 and 1,371,872.8 person‐years follow‐up, we found 11,824 incident depression cases and 10,051 incident anxiety cases, respectively. Compared with definite morning group, individuals with intermediate (hazard ratio [HR] = 1.09, 95% confidence interval [CI] = 1.04‒1.13) and definite evening chronotype (HR = 1.45, 95% CI = 1.36‒1.55) have higher risks of depression, and individuals with definite evening chronotype (HR = 1.27, 95% CI = 1.18‒1.37) have a higher risk of anxiety. The results were robust in the subgroup analysis of gender and age (Table S2), and in the sensitivity analysis (Table S3), which indicated that definite evening chronotype was related to higher risks of depression and anxiety compared with definite morning chronotype.

**TABLE 2:** Association of chronotype with depression and anxiety risk.

Chronotype	No. of depression cases (person‐years)	Depression, HR (95% CI)		No. of anxiety cases (person‐years)	Anxiety, HR (95% CI)	
		Model 1	Model 2		Model 1	Model 2
Definite morning chronotype	2999/593,828.1	Ref	Ref	2733/596,499.5	Ref	Ref
Intermediate chronotype	7455/1,399,716	1.07 (1.02‒1.12)	1.09 (1.04‒1.13)	6251/596,499.5	0.99 (0.94‒1.03)	1.00 (0.96‒1.05)
Definite evening chronotype	1370/1,399,716	1.51 (1.41‒1.60)	1.45 (1.36‒1.55)	1067/178,873.8	1.27 (1.19‒1.37)	1.27 (1.18‒1.37)

*Note*: Model 1—unadjusted. Model 2—adjusted for age, gender, country, education level, employment status, household income, and Townsend deprivation index.

Abbreviations: CI, confidence interval; HR, hazard ratio.

### 2.3 Joint Association of Chronotype and Genetic Risk with Risk of Depression/Anxiety

**FIGURE 1:**
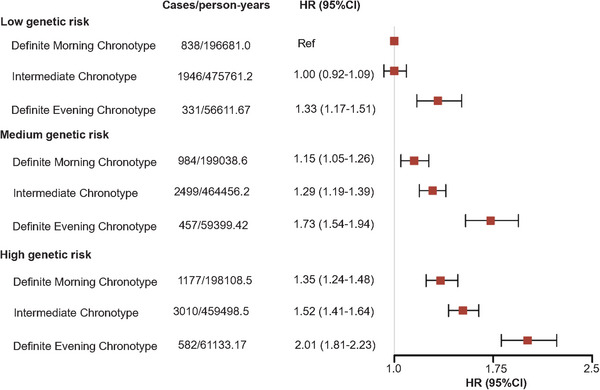
Joint association of chronotype and genetic risk with depression.

**FIGURE 2:**
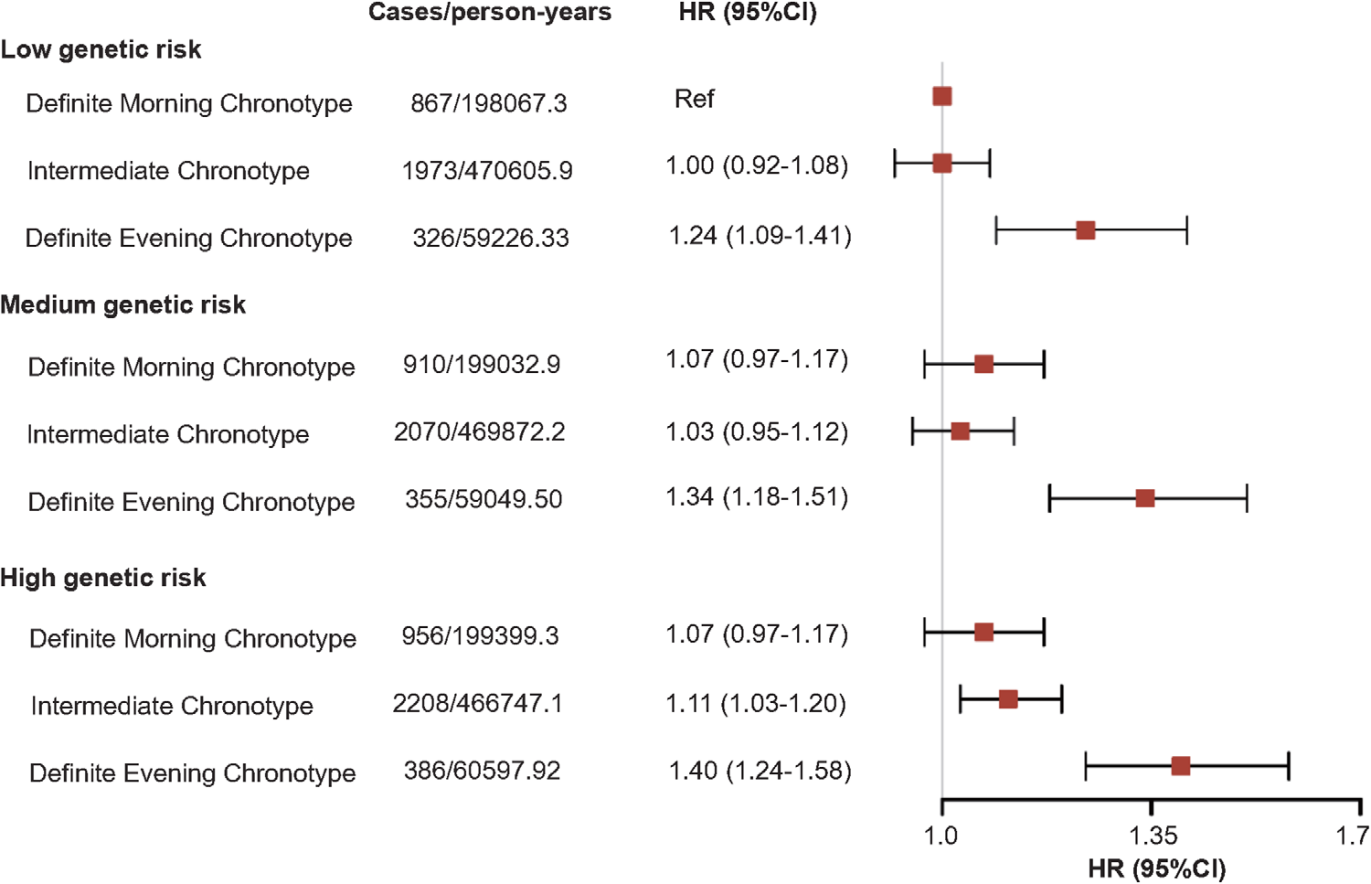
Joint association of chronotype and genetic risk with anxiety.

Table S4 reveals the relationship of genetic risk with depression and anxiety risk. Both continuous and category PRS are positively associated with depression and anxiety. Compared with low genetic risk, the HRs (95% CIs) for medium risk and high risk were 1.25 (1.20‒1.31) and 1.48 (1.41‒1.55) for depression, and 0.95 (0.91‒1.00) and 1.05 (1.01‒1.11) for anxiety, respectively. In addition, we explored the joint association of chronotype and genetic risk (Figures 1 and 2), and found individuals with definite evening chronotype and high genetic risk showed the highest risk of depression (HR = 2.01, 95% CI = 1.81‒2.23) and anxiety (HR = 1.40, 95% CI = 1.24‒1.58), when compared with individuals with low genetic risk and definite morning chronotype. The interaction between chronotype and genetic risk was not statistically significant for depression (*p*_interaction_ = 0.202) and anxiety (*p*_interaction_ = 0.770). The results were similar in the subgroup analysis of gender and age (Table S5), and in the sensitivity analysis (Figures S1–S5).

### 2.4 Joint Association of Chronotype and Lifestyle with Risk of Depression/Anxiety

**FIGURE 3:**
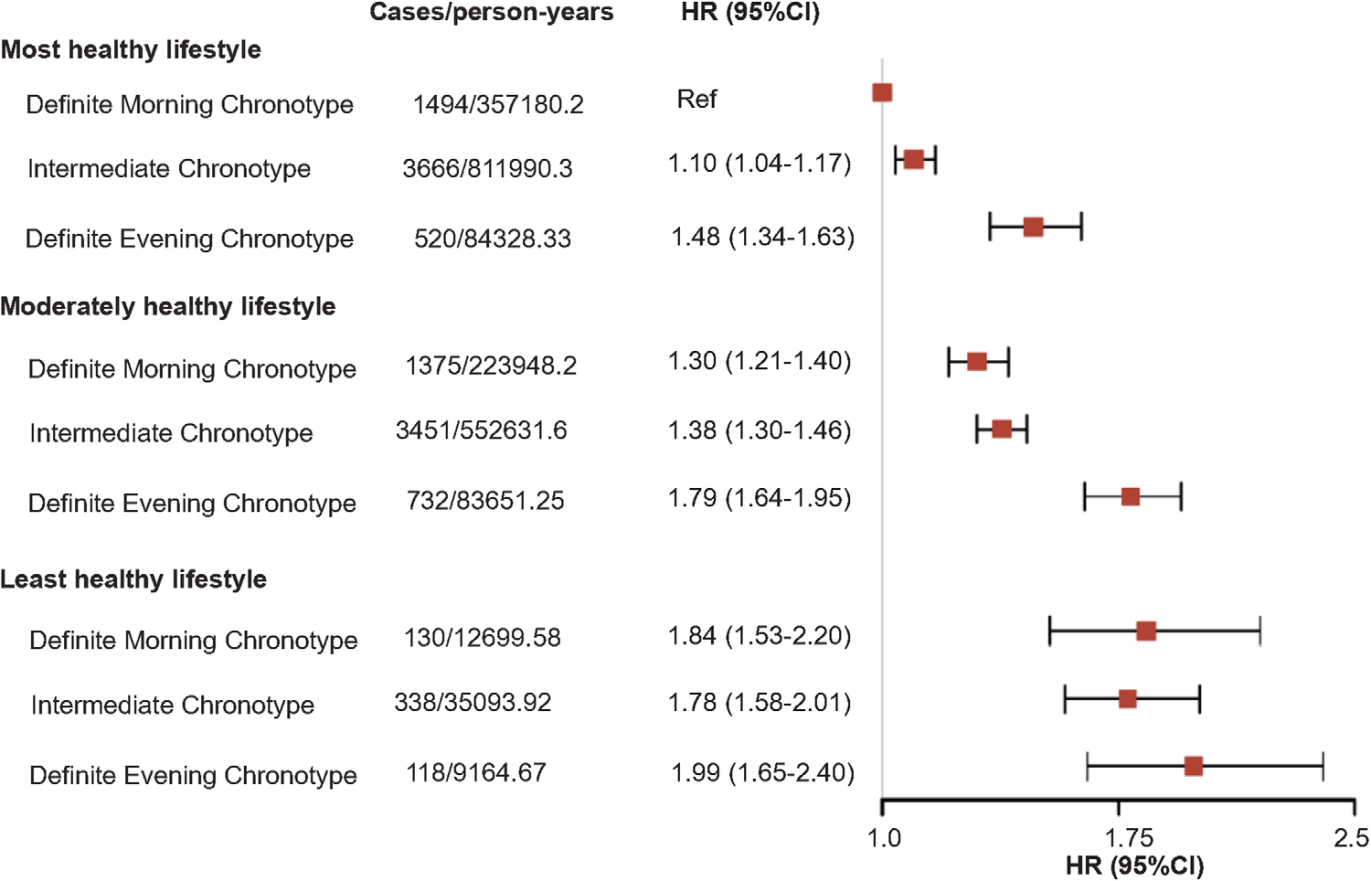
Joint association of chronotype and lifestyle with depression.

**FIGURE 4:**
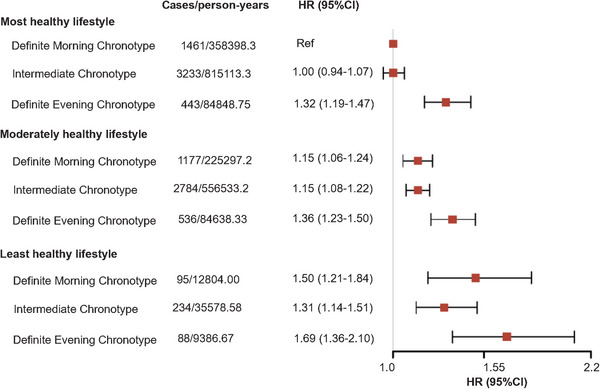
Joint association of chronotype and lifestyle with anxiety.

Table S6 shows the relationship of lifestyle with depression and anxiety risk. Compared with most healthy lifestyle, individuals with moderately healthy lifestyle and least healthy lifestyle had higher risks of depression (HR = 1.27, 95% CI = 1.23‒1.32 for moderately; HR = 1.66, 95% CI = 1.52‒1.82 for least) and anxiety (HR = 1.15, 95% CI = 1.10‒1.19 for moderately; HR = 1.39, 95% CI = 1.25‒1.54 for least). And the results were similar in specific lifestyle factors (Table S7). In addition, we explored the joint association of chronotype and lifestyle (Figures 3 and 4), and found those with definite evening chronotype and least healthy lifestyle showed the highest risk of depression (HR = 1.99, 95% CI = 1.65‒2.40) and anxiety (HR = 1.69, 95% CI = 1.36‒2.10), when compared with individuals with most healthy lifestyle and definite morning chronotype. The interaction between chronotype and lifestyle was not statistically significant for depression (*p*_interaction_ = 0.226) and anxiety (*p*_interaction_ = 0.466). The results were similar in the subgroup analysis of gender and age (Table S8), and in the sensitivity analysis (Figures S6‒S10). In addition, as chronotype may be associated with lifestyle, we conducted a mediating analysis of lifestyle in the association of chronotype with depression and anxiety (Table S9), and found that lifestyle played a mediating role in the association of chronotype with depression and anxiety, the mediating proportion ranged from 14.12% to 14.70%. As to specific lifestyle factors (Tables S10 and S11), smoking and physical activity play more important roles in the associations, the mediating proportion ranged from 4.93% to 9.40%.

We also conducted several sensitivity analyses when sleep duration was not included in the definition of lifestyle, and it was adjusted in the covariates. All the results were similar as before. The association of lifestyle with depression and anxiety, chronotype with incident depression and anxiety by lifestyle factors, subgroup analyses for association of lifestyle with depression and anxiety are presented in Tables S12‒S14, respectively. In addition, we found a joint association of chronotype and lifestyle with depression and anxiety (Figures S11 and S12). The interaction between chronotype and lifestyle was not statistically significant for depression (*p*_interaction_ = 0.081) and anxiety (*p*_interaction_ = 0.265). And we also found a mediating role of lifestyle in the association of chronotype with depression and anxiety (Tables S15‒S17).

## 3 Discussion

In our study, we found that individuals with intermediate and definite evening chronotype showed higher risks of depression, and individuals with definite evening chronotype showed a higher risk of anxiety. Furthermore, joint association was found between chronotype and genetic risk/lifestyle on depression and anxiety, those with definite evening chronotype and high genetic risk/least healthy lifestyle got the highest risk of depression and anxiety. In addition, lifestyle may play an important role in the association of chronotype with depression and anxiety.

Some limited previous studies have explored the association of chronotype with depression and anxiety; however, the results were inconsistent [16, 17, 18]. Vetter et al. found that chronotype was associated with a higher risk of depression among a women cohort [18]. However, Druiven et al. found that a later chronotype could not predict a persistent course of anxiety and depression at a 4‐year follow‐up [17]. They just found that change in chronotype was related to change in depression with a 7‐year follow‐up study, but not with anxiety [16]. Several factors may contribute to this phenomenon. For instance, inconsistency in definition and category of chronotype, the difference in study design, sample size, study population, etc. Vetter conducted the study among middle‐ and older‐aged women, and Druiven conducted study among depression and anxiety patients and controls to assess the relationship between chronotype and change in mental disorders. In addition, a recent study [19] has discussed the extant studies on the association between chronotype and mental health, and proposed that future cohort studies with large sample size and concrete basic information should be conducted to determine the casual relationship between chronotype and mental health. Previous study lacked evidence of large‐scale total population. Our study was conducted among 0.2 million populations including both sexes with a long follow‐up cohort study to assess the relationship of chronotype with depression and anxiety risk. We have adjusted the potential covariates and conducted subgroup analysis and several sensitivity analyses to verify our results, which could provide reliable conclusions. In our study, the follow‐up person‐years between depression and anxiety were substantially different, which may be related to case definitions, data sources, or follow‐up endpoints. In addition, the results in some subgroup analyses were shown with wider confidence intervals, which may be related to limited statistical power.

We have innovatively explored the joint association of chronotype with genetic risk and lifestyle on depression and anxiety, which has less been studied before. Limited studies were conducted from the National FINRISK study, but they were cross‐sectional study designs with relatively small study samples [20, 21, 22]. For genetic risk, they assessed the genetic association of chronotype [23], not for the genetic risk of mental health, and did not evaluate the interaction of chronotype and genetic risk on mental health either. Regarding lifestyle, they just evaluated the association of chronotype with separate lifestyles [24], including diet, obesity, etc. In our study, lifestyle was assessed comprehensively, which took more factors into account. We found that individuals with definite evening chronotype and high genetic risk got the highest risk of depression and anxiety, indicating that identifying high genetic risk is also useful in the prevention of depression and anxiety, especially for those with evening chronotype. Thus, some measures should be implemented to encourage residents adhering to a definite morning chronotype, for instance, the government could promote the benefits of definite morning chronotype and disadvantages of definite evening chronotype through news media or lectures. Meanwhile, more attention should be paid to families with depression and anxiety patients, which may have higher genetic risk. Furthermore, we also found that individuals with definite evening chronotype and least healthy lifestyle got the highest depression and anxiety risk. The results indicated that lifestyle is also significant in the association of chronotype with depression and anxiety. As a modified risk factor, change of unhealthy lifestyle may benefit individuals greatly with evening chronotype, especially for smoking, physical activity, television (TV) viewing time, fruit and vegetable intake, and oil fish intake. All these could contribute to the prevention and control of depression and anxiety in the early stage. In addition, previous studies indicated that the association of chronotype with depression and anxiety risk may be confused by sleep [25]; however, the positive association of evening chronotype with depression and anxiety could be found in both healthy and unhealthy sleep duration groups, which confirmed the robust of the results.

The underlying mechanism between evening chronotype and the risk of depression and anxiety is largely unknown. Some literatures hold that later chronotype leads to circadian rhythm disruption owing to a mismatch with work schedules [26], which could result in brain disorders and mental disorders [27]. Circadian rhythm was set by regulating neuronal activity, body temperature and hormonal signals under the suprachiasmatic nucleus [28]. Environmental factors such as stress could also alter circadian rhythm [27]. Furthermore, mental disorders associated with circadian disruption may also refer to endocrine and molecular mechanism. For instance, a loss of circadian rhythms gene expression may accompany delay in melatonin and cortisol rhythms [29]. And abnormal melatonin and cortisol rhythms could be found in mental disorder individuals [30]. In addition, studies revealed that evening chronotype was associated with state‐level impulsivity [25], and there may be some trait‐like aspect of chronotype, which is related with mental disorders. For instance, studies revealed that chronotype and risk of mental disorders or cannabis use shared similar genetic variance [31]. All these potential mechanisms need to be confirmed in future studies.

The present study has several strengths. First, it was a prospective cohort study with long‐term follow‐up time and large sample size. Second, the PRS was constructed by us using PRScs method, which could provide more accurate results. Some limitations should also be acknowledged. Chronotype was evaluated using a single question and was self‐reported, which may induce measurement error. However, it has been widely used in previous studies [32, 33], and studies have indicated that the consistent between the representative question and the overall Morningness‒Eveningness Questionnaire is well [23]. Moreover, it should be cautious when generalizing the findings, as the population in our study was all from the UK and the genetic risk scores are specific to European ancestry. Besides, several participants were excluded with missing information (Table S18), and some unmeasured confounding factors were not included, such as sleep quality or life stressors, all those may influence the results. Meanwhile, chronotype was just measured at baseline, without accounting for changes over time, which need to be confirmed in future studies. Furthermore, the identification of depression and anxiety relies solely on inpatient records and International Classification of Diseases, 10th Revision (ICD‐10) codes, which may miss cases diagnosed only in outpatient or community settings, and underestimate the true incidence. Moreover, other dietary behaviors were not included in the definition of lifestyle, which need to be confirmed in future. Finally, it is just an observational study, causal relationships cannot be definitively inferred, and the results need to be updated with updated data of UKB in future studies.

In conclusion, evening chronotype are associated with higher risks of depression and anxiety. Genetic risk and lifestyle are jointly associated with chronotype on the risk of depression and anxiety. Meanwhile, lifestyle may play an important role in the association of chronotype with depression and anxiety. It is necessary to encourage adults to adhere to a morning chronotype, especially for those with high genetic risk and unhealthy lifestyle. Future interventional or mechanistic research is needed to confirm the findings.

## 4 Materials and Methods

### 4.1 Study Population

UKB constitutes a large longitudinal cohort study, recruiting approximately 500,000 participants aged 40–69 years in the United Kingdom between 2006 and 2010. The information was gathered through standardized touchscreen questionnaires, physical measurement, health records, and biological samples. The details of the study could be found in a previous study [34].

We excluded those with depression or anxiety at baseline (*n* = 6379), without chronotype at baseline (*n* = 58,312), then excluded those with missing genetic data (*n* = 77,975), and missing information on lifestyle (*n* = 118,146); finally, we included 242,391 participants for the association of chronotype, genetic susceptibility, and lifestyle with the incidence of depression and anxiety (application number 88159). The flowchart could be found in Figure S13.

### 4.2 Chronotype

Chronotype was collected with touchscreen question. Participants will be asked “Do you consider yourself to be” (field ID: 1180 in UKB). And it has six responses: definitely an ‘evening’ person, more an ‘evening’ than a ‘morning’ person, more a ‘morning’ than ‘evening’ person, definitely a ‘morning’ person, do not know, and prefer not to answer. To reduce misclassification, we divided “more a morning” and “more an evening” types into an “intermediate” group, which is similar to previous studies [6].

### 4.3 Identification of Depression and Anxiety

The identification of incident depression and anxiety relied on hospitalization records using the ICD‐10 code (F32‒F33, F40‒F43, Table S19) [35, 36], including both primary and secondary diagnoses (field ID: 41270 in UKB).

### 4.4 Polygenic Risk Score

Genetic risk for anxiety and depression was quantified using PRS. We used the imputed genotypes from UKB. Details of the design of the array, sample processing and quality control in the UKB has been reported [37, 38]. We extracted a European ancestry subset (408,812 individuals) including samples who self‐identified as white British (UKB data field 21000, coding 1001, 1002, and 1003) or had very similar genetic ancestry based on a principal components analysis of the genotypes (UKB data field 22006). The variants were excluded by using PLINK 2.0 [39], with the following criteria: minor allele frequency (MAF) <0.001, missing genotype rate >0.05, Hardy–Weinberg equilibrium test *p*‐value <1.0 × 10^−12^, or imputation accuracy score <0.3 [38, 40]. Finally, 1.2 million HapMap3 variants on GRCh37 were retained for the following study.

PRSs for anxiety and depression were constructed by ourselves using PRScs (v.1.0.0) [41], a Bayesian method that applies a continuous shrinkage prior on the SNP effects. We used the PRScs‐auto algorithm, which automatically estimates the global shrinkage parameter using only genome‐wide association study (GWAS) summary statistics, eliminating the need for a tuning dataset. The GWAS summary statistics of depression [42] and anxiety [43] were obtained from the Psychiatric Genomics Consortium (PGC, https://pgc.unc.edu/). The detailed information could be found in Table S20.

For anxiety, case‒control summary statistics were sourced from PMID 26754954, comprising 6,330,995 SNPs from 17,310 individuals. For depression, summary statistics were derived from PMID 29700475 (excluding 23andMe and UKB samples), encompassing 9,874,287 SNPs from 53,586 individuals. In both cases, PRScs‐auto was executed with hyperparameters set to *a* = 1 and *b* = 0.5 to ensure methodological consistency.

### 4.5 Lifestyle Score

A composite lifestyle index was developed using nine behavioral factors: smoking status, alcohol intake, physical activity, TV viewing time, sleep duration, dietary intake (fruit and vegetable intake, oily fish intake, and red and processed meat intake) [44, 45]. Unhealthy lifestyle categories were defined as follows: current smoker; daily or almost daily alcohol intake; physical activity <150 min/week moderate or <75 min/week; TV viewing time ≥4 h/day of television; sleep time <7 or >9 h/day; fruits and vegetables <400 g/day; oily fish <1 portion per week; red meat >3 portions per week; processed meat >1 portion per week. An unweighted summative score ranged from 0 to 9 (Table S21), with a higher score indicating greater health risk. Participants were also divided into three groups: most healthy (0‒2), moderately healthy (3‒5), and least healthy (6‒9) based on the score.

### 4.6 Covariates

The basic information including sociodemographic factors, physical measurements, and medical history was collected with touchscreen questionnaires. Covariates in the present study were assessed using directed acyclic graphs (DAGs) (Figure S14) with DAGitty version 3.1 software. DAGs could provide accurate estimates for causal inference and confounding control between the exposure and outcomes, and have been widely used in observational studies [46]. The covariates were selected according to published studies [6, 9] and must meet the criteria established in epidemiological studies. Finally, covariates included age, gender, country, education level, household income, employment status, and Townsend deprivation index. Missing information of continuous and categorical variables were imputed with sex‐specific median values or coded as a missing indicator category, respectively [47]. The detailed information on covariates could be found in Table S22.

### 4.7 Statistical Analysis

The analyses in our study were considered exploratory. Cox proportional hazards regression models were used to calculate HRs and 95% CIs for the association of chronotype, genetic risk, and lifestyle with depression and anxiety [6]. Follow‐up time was determined from date of recruitment until date of first incidence of depression or anxiety, loss to follow‐up, death, or the end of follow‐up (February 28, 2018), whichever came first [35]. We also conducted subgroup analysis and sensitivity analysis. We first excluded those cases in the first 2 years, then excluded anxiety cases when analysis depression and excluded depression cases when analysis anxiety. Besides, we added other covariates including hypertension, diabetes, BMI, and lifestyle score. Finally, we excluded missing covariates.

To assess the joint relationship of genetic risk and lifestyle with chronotype, the combined relationship of chronotype and genetic risk/lifestyle with depression and anxiety was also conducted. Furthermore, the multiplicative interactions between chronotype and genetic risk/lifestyle (chronotype × genetic risk, chronotype × lifestyle) were also tested. Finally, we assessed the mediating role of lifestyle in the relationship between chronotype and mental health using the “mediation” package. We also conducted several sensitivity analyses when sleep duration was not included in lifestyle, and we adjusted it in the covariates.

Data were analyzed using SAS 9.4 or R 4.0.5, and statistical significance was set as a two‐tailed *p*‐value <0.05.

## Author Contributions

**Dongming Wang**: conceptualization (lead), writing – original draft (lead), formal analysis (lead), writing – review and editing (equal). **Zhaomin Chen**: software (lead), writing – review and editing (equal). **Zhonghe Shao** and **Xingjie Hao**: methodology (lead), writing – review and editing (equal). **Wenzhen Li**: conceptualization (equal), writing – original draft (supporting), writing – review and editing (equal). Wenzhen Li are the guarantors of the present study. All the authors have read and approved the final manuscript.

## Funding

The study was supported by National Natural Science Foundation of China (42507573). The funder did not play any role in the present study.

## Ethics Statement

The ethics approval was authorized by the North West Multi‐Centre Research Ethics Committee (16/NW/0274). Written informed consent was obtained from all participants.

## Conflicts of Interest

The authors declare no conflicts of interest.

## Supporting information




**TABLE S1** Characteristics of participants at baseline by chronotype.
**TABLE S2** Subgroup analyses for association between chronotype and risk of depression and anxiety.
**TABLE S3** Sensitive analyses of chronotype in relation to depression and anxiety.
**TABLE S4** Association of genetic risk with incident depression and anxiety.
**TABLE S5** Subgroup analyses for association of genetic risk with depression and anxiety.
**TABLE S6** Association of lifestyle with incident depression and anxiety.
**TABLE S7** Association of chronotype with incident depression and anxiety by lifestyle factors.
**TABLE S8** Subgroup analyses for association of lifestyle with depression and anxiety.
**TABLE S9** Mediating role of lifestyle in the association of chronotype with depression and anxiety.
**TABLE S10** Mediating role of specific lifestyle factors in the association between chronotype and depression.
**TABLE S11** Mediating role of specific lifestyle factors in the association between chronotype and anxiety.
**TABLE S12** Sensitive analyses for association between lifestyle and incident depression and anxiety.
**TABLE S13** Sensitive analyses for association of chronotype with incident depression and anxiety by lifestyle factors.
**TABLE S14** Sensitive analyses of subgroup analyses for association of lifestyle with depression and anxiety.
**TABLE S15** Sensitive analyses for mediating role of lifestyle in the association of chronotype with depression and anxiety.
**TABLE S16** Sensitive analyses for mediating role of specific lifestyle factors in the association between chronotype and depression.
**TABLE S17** Sensitive analyses for mediating role of specific lifestyle factors in the association between chronotype and anxiety.
**TABLE S18** Characteristics of participants with (*n* = 242,391) and without (*n* = 118,146) lifestyle data.
**TABLE S19** ICD‐10 codes used to ascertain anxiety and depression in the UK Biobank.
**TABLE S20** Best‐fitting parameters of the polygenetic risk scores for depression and anxiety.
**TABLE S21** Variables used to create lifestyle score for UK Biobank.
**TABLE S22** Covariates definitions and assessment in our study.
**FIGURE S1** Sensitive analyses for the joint association of chronotype and genetic risk with depression and anxiety when excluding cases occurred in the first 2 years of follow‐up: (A) depression and (B) anxiety.
**FIGURE S2** Sensitive analyses for the joint association of chronotype and genetic risk with depression when excluding anxiety cases during follow‐up.
**FIGURE S3** Sensitive analyses for the joint association of chronotype and genetic risk with anxiety when excluding depression cases during follow‐up.
**FIGURE S4** Sensitive analyses for the joint association of chronotype and genetic risk with depression and anxiety when additional adjustment for other covariates including hypertension, diabetes, BMI, and lifestyle score: (A) depression and (B) anxiety.
**FIGURE S5** Sensitive analyses for the joint association of chronotype and genetic risk with depression and anxiety when excluding missing covariates: (A) depression and (B) anxiety.
**FIGURE S6** Sensitive analyses for the joint association of chronotype and lifestyle with depression and anxiety when excluding cases occurred in the first 2 years of follow‐up: (A) depression and (B) anxiety.
**FIGURE S7** Sensitive analyses for the joint association of chronotype and lifestyle with depression when excluding anxiety cases during follow‐up.
**FIGURE S8** Sensitive analyses for the joint association of chronotype and lifestyle with anxiety when excluding depression cases during follow‐up.
**FIGURE S9** Sensitive analyses for the joint association of chronotype and lifestyle with depression and anxiety when additional adjustment for other covariates including hypertension, diabetes, BMI, and PRS: (A) depression and (B) anxiety.
**FIGURE S10** Sensitive analyses for the joint association of chronotype and lifestyle with depression and anxiety when excluding missing covariates: (A) depression and (B) anxiety.
**FIGURE S11** Sensitive analyses for the joint association of chronotype and lifestyle with depression when sleep duration was not included in lifestyle.
**FIGURE S12** Sensitive analyses for the joint association of chronotype and lifestyle with anxiety when sleep duration was not included in lifestyle.
**FIGURE S13** Flowchart of the study.
**FIGURE S14** Directed acyclic graph for the association of chronotype with depression and anxiety.

## Data Availability

The data are available from the UK Biobank (https://www.ukbiobank.ac.uk/). It could be available with reasonable request from the corresponding author.
