# Supplementary material for: Chronotype, Genetic Risk, Lifestyle, and Risk of Depression and Anxiety: A Prospective Cohort Study
Source: MedComm (2020). 2026 Apr 8;7(4):e70736. doi: 10.1002/mco2.70736 (PMC13062636; doi:10.1002/mco2.70736)
Supplement: Supplementary file 1 — TABLE S1 Characteristics of participants at baseline by chronotype. TABLE S2 Subgroup analyses for association between chronotype and risk of depression and anxiety. TABLE S3 Sensitive analyses of chronotype in relation to depression and anxiety. TABLE S4 Association of genetic risk with incident depression and anxiety. TABLE S5 Subgroup analyses for association of genetic risk with depression and anxiety. TABLE S6 Association of lifestyle with incident depression and anxiety. TABLE S7 Association of chronotype with incident depression and anxiety by lifestyle factors. TABLE S8 Subgroup analyses for association of lifestyle with depression and anxiety. TABLE S9 Mediating role of lifestyle in the association of chronotype with depression and anxiety. TABLE S10 Mediating role of specific lifestyle factors in the association between chronotype and depression. TABLE S11 Mediating role of specific lifestyle factors in the association between chronotype and anxiety. TABLE S12 Sensitive analyses for association between lifestyle and incident depression and anxiety. TABLE S13 Sensitive analyses for association of chronotype with incident depression and anxiety by lifestyle factors. TABLE S14 Sensitive analyses of subgroup analyses for association of lifestyle with depression and anxiety. TABLE S15 Sensitive analyses for mediating role of lifestyle in the association of chronotype with depression and anxiety. TABLE S16 Sensitive analyses for mediating role of specific lifestyle factors in the association between chronotype and depression. TABLE S17 Sensitive analyses for mediating role of specific lifestyle factors in the association between chronotype and anxiety. TABLE S18 Characteristics of participants with (n = 242,391) and without (n = 118,146) lifestyle data. TABLE S19 ICD‐10 codes used to ascertain anxiety and depression in the UK Biobank. TABLE S20 Best‐fitting parameters of the polygenetic risk scores for depression and anxiety. TABLE S21 Variables used to create l [file MCO2-7-e70736-s001.docx]

**Chronotype, genetic risk, lifestyle and risk of depression and anxiety: a prospective cohort study**

Dongming Wang ^1, 2^, Zhonghe Shao ^3^, Zhaomin Chen ^1,2^, Xingjie Hao ^3^, Wenzhen Li ^1,4,5,*^

**Author Affiliations:**

^1^ Department of Occupational & Environmental Health, School of Public Health, Tongji Medical College, Huazhong University of Science and Technology, Wuhan, Hubei 430030, China

^2^ Key Laboratory of Environment and Health, Ministry of Education & Ministry of Environmental Protection, and State Key Laboratory of Environmental Health (Incubating), School of Public Health, Tongji Medical College, Huazhong University of Science and Technology, Wuhan, Hubei 430030, China

^3^ Department of Epidemiology and Biostatistics, School of Public Health, Tongji Medical College, Huazhong University of Science and Technology, Wuhan, Hubei 430030, China

^4^ Jockey Club School of Public Health and Primary Care, The Chinese University of Hong Kong, Hong Kong, China

^5^ Shenzhen Research Institute of the Chinese University of Hong Kong, Shenzhen, China

***Corresponding Author:**

**Wenzhen Li**

Jockey Club School of Public Health and Primary Care, The Chinese University of Hong Kong, Hong Kong, China

**E-mail:** [wenzhenli@cuhk.edu.hk](mailto:wenzhenli@cuhk.edu.hk)

**Table S1 Characteristics of participants at baseline by chronotype**

| **Characteristics** | **Definite morning chronotype (n=66,255)** | **Intermediate chronotype (n=156,206)** | **Definite evening chronotype (n=19,930)** |
| --- | --- | --- | --- |
| **Age, years (mean ± SD)** | 57.81 ± 7.70 | 56.67 ± 8.055 | 55.42±8.29 |
| **Gender** |  |  |  |
| Female | 36,199 (54.64) | 83,886 (53.07) | 10,316 (51.76) |
| Male | 30,056 (45.36) | 72,320 (46.30) | 9,614 (48.24) |
| **Country** |  |  |  |
| England | 63,194 (95.38) | 149,627 (95.79) | 18,935 (95.01) |
| Wales | 3,061 (4.62) | 6,579 (4.21) | 995 (4.99) |
| **Education** |  |  |  |
| Higher | 18,934 (28.58) | 49,304 (31.56) | 7,422 (37.24) |
| Upper secondary | 20,838 (31.45) | 55,396 (35.46) | 6,988 (35.06) |
| Lower secondary | 3,660 (5.52) | 8,779 (5.62) | 890 (4.47) |
| Vocational | 8,675 (13.09) | 18,783 (12.02) | 2,189 (10.98) |
| No secondary education | 13,683 (20.65) | 23,208 (14.86) | 2,358 (11.83) |
| Prefer not to answer | 465 (0.70) | 736 (0.47) | 83 (0.42) |
| **Household income** |  |  |  |
| Less than £31,000 | 29,488 (44.51) | 64,385 (41.22) | 8,265 (41.47) |
| £31,000 and above | 28,817 (43.49) | 75,504 (48.34) | 9,742 (48.88) |
| Missing data | 7,950 (12.00) | 16,317 (10.45) | 1,923 (9.65) |
| **Townsend deprivation index (mean ± SD)** | -1.58 ± 2.91 | -1.73 ± 2.83 | -1.25 ± 3.07 |
| **Current employment status** |  |  |  |
| Employed | 36,387 (54.92) | 90,424 (57.89) | 11,895 (59.68) |
| Retired | 25,289 (38.17) | 54,591 (34.95) | 5,927 (29.74) |
| Unemployed, home | 4,171 (6.30) | 10,374 (6.64) | 1,976 (9.91) |
| None of the above | 269 (0.41) | 580 (0.37) | 86 (0.43) |
| Prefer not to answer | 139 (0.21) | 237 (0.15) | 46 (0.23) |
| **Healthy lifestyle factor** |  |  |  |
| Smoking | 5,229 (7.89) | 14,511 (9.29) | 3,425 (17.19) |
| Alcohol intake | 13,111 (19.79) | 34,259 (21.93) | 4,940 (24.79) |
| Physical activity | 29,835 (45.03) | 73,240 (46.89) | 10,607 (53.22) |
| TV viewing | 19,740 (29.79) | 45,476 (29.11) | 6,220 (31.21) |
| Sleep time | 18,704 (28.23) | 35,577 (22.78) | 5,981 (30.01) |
| Fruit and vegetable intake | 9,235 (13.94) | 25,334 (16.22) | 4,331 (21.73) |
| Oily fish intake | 26,567 (40.10) | 66,381 (42.50) | 9,156 (45.94) |
| Red meat intake | 9,217 (13.91) | 21,717 (13.90) | 3,110 (15.60) |
| Processed meat intake | 18,517 (27.95) | 49,762 (31.86) | 7,129 (35.77) |
| **Lifestyle category** |  |  |  |
| Most healthy | 39,681 (59.89) | 90,299 (57.81) | 9,421 (47.27) |
| Moderately healthy | 25,109 (37.90) | 61,899 (39.63) | 9,442 (47.38) |
| Least healthy | 1,465 (2.21) | 4,008 (2.57) | 1,067 (5.35) |
| **Genetic risk category** |  |  |  |
| Depression |  |  |  |
| Low | 21,910 (33.07) | 52,935 (33.89) | 6,333 (31.78) |
| Medium | 22,202 (33.51) | 51,838 (33.19) | 6,690 (33.57) |
| High | 22,143 (33.42) | 51,433 (32.93) | 6,907 (34.66) |
| Anxiety |  |  |  |
| Low | 21,999 (33.20) | 52,201 (33.42) | 6,597 (33.10) |
| Medium | 22,103 (33.36) | 52,123 (33.37) | 6,571 (32.97) |
| High | 22,153 (33.44) | 51,882 (33.21) | 6,762 (33.93) |
| **BMI (mean ± SD)** | 27.52 ± 4.721 | 27.25 ± 4.584 | 27.93 ± 5.032 |
| **Hypertension** | 19,193 (28.97) | 41,809 (26.77) | 5,330 (26.74) |
| **Diabetes** | 3,371 (5.09) | 7,117 (4.56) | 1,236 (6.20) |

Continues variables are displayed as means ± SD, and categorical variables are displayed as numbers (percentages).

Abbreviations: SD, standard deviation, BMI, body mass index.

**Table S2 Subgroup analyses for association between chronotype and risk of depression and anxiety**

| **Chronotype** | **Definite morning chronotype** | **Intermediate chronotype** | |  | **Definite evening chronotype** | |
| --- | --- | --- | --- | --- | --- | --- |
|  |  | **Depression** | **Anxiety** |  | **Depression** | **Anxiety** |
| **Female** |  |  |  |  |  |  |
| Model 1 | Ref | 1.12 (1.06-1.18) | 1.01 (0.96-1.67) |  | 1.51 (1.39-1.63) | 1.24 (1.14-1.36) |
| Model 2 | Ref | 1.13 (1.07-1.19) | 1.03 (0.97-1.08) |  | 1.46 (1.34-1.58) | 1.27 (1.16-1.39) |
| **Male** |  |  |  |  |  |  |
| Model 1 | Ref | 1.00 (0.93-1.07) | 0.96 (0.88-1.03) |  | 1.51 (1.37-1.68) | 1.32 (1.18-1.49) |
| Model 2 | Ref | 1.01 (0.99-1.09) | 0.96 (0.89-1.04) |  | 1.43 (1.29-1.59) | 1.24 (1.10-1.40) |
| **Age <60** |  |  |  |  |  |  |
| Model 1 | Ref | 1.05 (0.99-1.11) | 0.97 (0.91-1.03) |  | 1.46 (1.34-1.58) | 1.34 (1.22-1.47) |
| Model 2 | Ref | 1.08 (1.01-1.14) | 0.97 (0.91-1.04) |  | 1.42 (1.30-1.54) | 1.33 (1.21-1.47) |
| **Age ≥60** |  |  |  |  |  |  |
| Model 1 | Ref | 1.08 (1.10-1.14) | 1.01 (0.95-1.08) |  | 1.51 (1.36-1.66) | 1.17 (1.04-1.30) |
| Model 2 | Ref | 1.11 (1.05-1.18) | 1.04 (0.98-1.11) |  | 1.53 (1.38-1.69) | 1.17 (1.05-1.31) |

Adjusted for age, gender, country, education level, employment status, household income, and Townsend deprivation index.

**Table S3** **Sensitive analyses of chronotype in relation to depression and anxiety**

|  | **Definite morning chronotype** | **Intermediate chronotype** | **Definite evening chronotype** |
| --- | --- | --- | --- |
| **Sensitive 1** |  |  |  |
| Depression (n=240,316) | Ref | 1.08 (1.03-1.13) | 1.43 (1.34-1.53) |
| Anxiety (n=240,763) | Ref | 1.00 (0.96-1.05) | 1.27 (1.18-1.36) |
| **Sensitive 2** |  |  |  |
| Depression (n=232,340) | Ref | 1.10 (1.04-1.16) | 1.48 (1.37-1.60) |
| **Sensitive 3** |  |  |  |
| Anxiety (n=230,567) | Ref | 0.97 (0.92-1.03) | 1.19 (1.09-1.31) |
| **Sensitive 4** |  |  |  |
| Depression (n=242,391) | Ref | 1.08 (1.03-1.13) | 1.36 (1.28-1.45) |
| Anxiety (n=242,391) | Ref | 1.00 (0.95-1.04) | 1.23 (1.14-1.32) |
| **Sensitive 5** |  |  |  |
| Depression (n=215,639) | Ref | 1.07 (1.02-1.12) | 1.44 (1.34-1.54) |
| Anxiety (n=215,639) | Ref | 0.99 (0.94-1.04) | 1.26 (1.17-1.36) |

HR: hazard ratio; CI: confidence interval.

Adjusted for age, gender, country, education level, employment status, household income, and Townsend deprivation index.

Sensitive1: Excluding cases occurred in the first 2 years of follow-up.

Sensitive 2: Excluding anxiety cases during follow-up.

Sensitive 3: Excluding depression cases during follow-up.

Sensitive 4: Additional adjustment for other covariates including hypertension, diabetes, bmi, lifestyle score.

Sensitive 5: Excluding missing covariates.

**Table S4 Association of genetic risk with incident depression and anxiety**

|  | **Depression** | | **Anxiety** | |
| --- | --- | --- | --- | --- |
|  | **Cases/person-years** | **HR (95% CI)** | **Cases/person-years** | **HR (95% CI)** |
| Continuous | 11,824/2,170,688 | 2.05 (1.90-2.21) | 10,051/2,182,598 | 1.15 (1.08-1.22) |
| **Categories of PRS according to tertile** |  |  |  |  |
| Low (tertile 1) | 3,115/729,053.8 | Ref | 3,166/727,899.5 | Ref |
| Medium (tertile 2) | 3,940/722,894.2 | 1.25 (1.20-1.31) | 3,335/727,954.6 | 0.95 (0.91-1.00) |
| High (tertile 3) | 4,769/718,740.2 | 1.48 (1.41-1.55) | 3,550/726,744.3 | 1.05 (1.01-1.11) |

Adjusted for age, gender, country, education level, employment status, household income, and Townsend deprivation index.

**Table S5 Subgroup analyses for association of genetic risk with depression and anxiety**

|  | **HR (95% CI)** | |
| --- | --- | --- |
|  | **Depression** | **Anxiety** |
| **Female** |  |  |
| Continuous | 2.11 (1.92-2.32) | 1.15 (1.07-1.24) |
| Low (tertile 1) | Ref | Ref |
| Medium (tertile 2) | 1.26 (1.19-1.34) | 1.07 (1.01-1.14) |
| High (tertile 3) | 1.51 (1.43-1.60) | 1.10 (1.04-1.17) |
| **Male** |  |  |
| Continuous | 1.94 (1.71-2.20) | 1.14 (1.02-1.26) |
| Low (tertile 1) | Ref | Ref |
| Medium (tertile 2) | 1.23 (1.14-1.33) | 1.01 (0.93-1.09) |
| High (tertile 3) | 1.42 (1.31-1.52) | 1.11 (1.02-1.20) |
| **Age <60** |  |  |
| Continuous | 2.10 (1.90-2.33) | 1.14 (1.05-1.24) |
| Low (tertile 1) | Ref | Ref |
| Medium (tertile 2) | 1.24 (1.16-1.32) | 1.05 (0.98-1.12) |
| High (tertile 3) | 1.47 (1.39-1.57) | 1.09 (1.02-1.17) |
| **Age ≥60** |  |  |
| Continuous | 1.98 (1.77-2.21) | 1.13 (1.04-1.23) |
| Low (tertile 1) | Ref | Ref |
| Medium (tertile 2) | 1.28 (1.19-1.37) | 1.04 (0.97-1.11) |
| High (tertile 3) | 1.48 (1.38-1.58) | 1.10 (1.03-1.18) |

Adjusted for age, gender, country, education level, employment status, household income, and Townsend deprivation index.

**Table S6 Association of lifestyle with incident depression and anxiety**

|  | **HR (95% CI)** | | |
| --- | --- | --- | --- |
|  | **Depression** | **Anxiety** |  |
| **Lifestyle score** |  |  |  |
| 0 (Most healthy) | Ref | Ref |  |
| 1 | 1.09 (1.01-1.19) | 1.05 (0.97-1.15) |  |
| 2 | 1.23 (1.14-1.33) | 1.16 (1.07-1.27) |  |
| 3 | 1.37 (1.26-1.48) | 1.22 (1.12-1.32) |  |
| 4 | 1.52 (1.39-1.65) | 1.28 (1.17-1.40) |  |
| 5 | 1.66 (1.50-1.82) | 1.37 (1.24-1.52) |  |
| 6 | 1.85 (1.64-2.09) | 1.44 (1.25-1.65) |  |
| ≥7 (Least healthy) | 2.14 (1.80-2.53) | 1.83 (1.51-2.22) |  |
| **Lifestyle category** |  |  |  |
| Most healthy | Ref | Ref |  |
| Moderately healthy | 1.27 (1.23-1.32) | 1.15 (1.10-1.19) |  |
| Least healthy | 1.66 (1.52-1.82) | 1.39 (1.25-1.54) |  |
| **Lifestyle factors** |  |  |  |
| Smoking status | 1.37 (1.30-1.44) | 1.15 (1.09-1.22) |  |
| Alcohol intake | 1.00 (0.95-1.05) | 1.00 (0.95-1.05) |  |
| Physical activity | 1.22 (1.17-1.26) | 1.11 (1.07-1.16) |  |
| Television viewing time | 1.16 (1.12-1.21) | 1.12 (1.07-1.17) |  |
| Sleep duration | 1.39 (1.34-1.45) | 1.28 (1.23-1.34) |  |
| Fruit and vegetable intake | 1.17 (1.12-1.23) | 1.15 (1.09-1.21) |  |
| Oily fish intake | 1.07 (1.03-1.11) | 1.06 (1.02-1.11) |  |
| Red meat intake | 0.92 (0.87-1.03) | 0.87 (0.80-1.02) |  |
| Processed meat intake | 1.09 (1.05-1.14) | 1.07 (1.02-1.11) |  |

HR: hazard ratio; CI: confidence interval.

Adjusted for age, gender, country, education level, employment status, household income, and Townsend deprivation index.

**Table S7 Association of chronotype with incident depression and anxiety by lifestyle factors**

| **Lifestyle factors** | **HR (95% CI)** | |
| --- | --- | --- |
|  | **Depression** | **Anxiety** |
| **Smoking status (healthy)** |  |  |
| Definite morning chronotype | Ref | Ref |
| Intermediate chronotype | 1.11 (1.06-1.16) | 1.02 (0.97-1.07) |
| Definite evening chronotype | 1.51 (1.40-1.62) | 1.27 (1.17-1.39) |
| **Smoking status (unhealthy)** |  |  |
| Definite morning chronotype | Ref | Ref |
| Intermediate chronotype | 0.92 (0.82-1.03) | 0.87 (0.76-0.99) |
| Definite evening chronotype | 1.07 (0.92-1.23) | 1.12 (0.95-1.32) |
| **Alcohol intake (healthy)** |  |  |
| Definite morning chronotype | Ref | Ref |
| Intermediate chronotype | 1.10 (1.05-1.15) | 1.02 (0.97-1.07) |
| Definite evening chronotype | 1.47 (1.36-1.58) | 1.30 (1.20-1.41) |
| **Alcohol intake (unhealthy)** |  |  |
| Definite morning chronotype | Ref | Ref |
| Intermediate chronotype | 1.04 (0.94-1.15) | 0.94 (0.85-1.05) |
| Definite evening chronotype | 1.40 (1.21-1.61) | 1.18 (1.01-1.38) |
| **Physical activity (healthy)** |  |  |
| Definite morning chronotype | Ref | Ref |
| Intermediate chronotype | 1.08 (1.02-1.15) | 0.99 (0.92-1.05) |
| Definite evening chronotype | 1.46 (1.32-1.61) | 1.34 (1.21-1.49) |
| **Physical activity (unhealthy)** |  |  |
| Definite morning chronotype | Ref | Ref |
| Intermediate chronotype | 1.08 (1.02-1.24) | 1.01 (0.95-1.07) |
| Definite evening chronotype | 1.41 (1.30-1.54) | 1.20 (1.09-1.32) |
| **TV viewing time (healthy)** |  |  |
| Definite morning chronotype | Ref | Ref |
| Intermediate chronotype | 1.08 (1.02-1.14) | 0.98 (0.93-1.04) |
| Definite evening chronotype | 1.41 (1.30-1.53) | 1.25 (1.14-1.36) |
| **TV viewing time (unhealthy)** |  |  |
| Definite morning chronotype | Ref | Ref |
| Intermediate chronotype | 1.09 (1.01-1.17) | 1.03 (0.96-1.11) |
| Definite evening chronotype | 1.48 (1.33-1.64) | 1.28 (1.14-1.43) |
| **Sleep duration (healthy)** |  |  |
| Definite morning chronotype | Ref | Ref |
| Intermediate chronotype | 1.10 (1.05-1.16) | 1.02 (0.97-1.08) |
| Definite evening chronotype | 1.50 (1.38-1.62) | 1.35 (1.23-1.47) |
| **Sleep duration (unhealthy)** |  |  |
| Definite morning chronotype | Ref | Ref |
| Intermediate chronotype | 1.11 (1.03-1.19) | 0.99 (0.92-1.08) |
| Definite evening chronotype | 1.36 (1.23-1.51) | 1.13 (1.00-1.27) |
| **Fruit and vegetable intake (healthy)** |  |  |
| Definite morning chronotype | Ref | Ref |
| Intermediate chronotype | 1.10 (1.05-1.15) | 1.00 (0.95-1.05) |
| Definite evening chronotype | 1.49 (1.39-1.60) | 1.23 (1.13-1.33) |
| **Fruit and vegetable intake (unhealthy)** | |  |
| Definite morning chronotype | Ref | Ref |
| Intermediate chronotype | 1.01 (0.91-1.12) | 1.01 (0.91-1.14) |
| Definite evening chronotype | 1.25 (1.09-1.44) | 1.37 (1.18-1.60) |
| **Oily fish intake (healthy)** |  |  |
| Definite morning chronotype | Ref | Ref |
| Intermediate chronotype | 1.12 (1.06-1.18) | 1.00 (0.94-1.06) |
| Definite evening chronotype | 1.51 (1.38-1.64) | 1.24 (1.12-1.37) |
| **Oily fish intake (unhealthy)** |  |  |
| Definite morning chronotype | Ref | Ref |
| Intermediate chronotype | 1.04 (0.98-1.11) | 1.00 (0.93-1.07) |
| Definite evening chronotype | 1.38 (1.26-1.52) | 1.30 (1.17-1.44) |
| **Red meat intake (healthy)** |  |  |
| Definite morning chronotype | Ref | Ref |
| Intermediate chronotype | 1.10 (1.05-1.15) | 1.02 (0.97-1.07) |
| Definite evening chronotype | 1.50 (1.40-1.61) | 1.32 (1.22-1.42) |
| **Red meat intake (unhealthy)** |  |  |
| Definite morning chronotype | Ref | Ref |
| Intermediate chronotype | 1.00 (0.89-1.12) | 0.91 (0.81-1.03) |
| Definite evening chronotype | 1.23 (1.04-1.46) | 1.05 (0.86-1.27) |
| **Processed meat intake (healthy)** |  |  |
| Definite morning chronotype | Ref | Ref |
| Intermediate chronotype | 1.12 (1.06-1.17) | 1.02 (0.97-1.08) |
| Definite evening chronotype | 1.49 (1.38-1.61) | 1.31 (1.20-1.43) |
| **Processed meat intake (unhealthy)** |  |  |
| Definite morning chronotype | Ref | Ref |
| Intermediate chronotype | 1.01 (0.93-1.09) | 0.94 (0.87-1.02) |
| Definite evening chronotype | 1.35 (1.21-1.51) | 1.17 (1.03-1.32) |

HR: hazard ratio; CI: confidence interval.

Adjusted for age, gender, country, education level, employment status, household income, and Townsend deprivation index.

**Table S8 Subgroup analyses for association of lifestyle with depression and anxiety**

|  | **HR (95% CI)** | |
| --- | --- | --- |
|  | **Depression** | **Anxiety** |
| **Female** |  |  |
| Most healthy | Ref | Ref |
| Moderately healthy | 1.33 (1.27-1.39) | 1.15 (1.10-1.21) |
| Least healthy | 1.84 (1.61-2.10) | 1.52 (1.31-1.77) |
| **Male** |  |  |
| Most healthy | Ref | Ref |
| Moderately healthy | 1.16 (1.09-1.24) | 1.12 (1.04-1.20) |
| Least healthy | 1.47 (1.30-1.66) | 1.26 (1.09-1.46) |
| **Age <60** |  |  |
| Most healthy | Ref | Ref |
| Moderately healthy | 1.34 (1.27-1.41) | 1.19 (1.12-1.26) |
| Least healthy | 1.73 (1.55-1.93) | 1.40 (1.23-1.59) |
| **Age ≥60** |  |  |
| Most healthy | Ref | Ref |
| Moderately healthy | 1.21 (1.14-1.28) | 1.11 (1.05-1.18) |
| Least healthy | 1.54 (1.33-1.78) | 1.28 (1.08-1.51) |

Adjusted for age, gender, country, education level, employment status, household income, and Townsend deprivation index.

**Table S9 Mediating role of lifestyle in the association of chronotype with depression and anxiety**

| **Outcome** | **Chronotype** | **a** | **b** | **Total effect** | **Direct effect** | **Indirect effect** | **Proportion, %** |
| --- | --- | --- | --- | --- | --- | --- | --- |
| Anxiety | Definite morning chronotype | Ref | 1.10 (1.08, 1.11) |  |  |  |  |
|  | Definite evening chronotype | 0.49 (0.47, 0.51) |  | 1.87 (1.84, 2.31) | 1.60 (1.57, 2.02) | 0.27 (0.25, 0.31) | 14.62** |
| Depression | Definite morning chronotype | Ref | 1.13 (1.12, 1.14) |  |  |  |  |
|  | Intermediate chronotype | 0.10 (0.09, 0.12) |  | 0.50 (0.31, 0.74) | 0.42 (0.24, 0.65) | 0.07 (0.06, 0.09) | 14.70** |
|  | Definite evening chronotype | 0.49 (0.47, 0.51) |  | 2.85 (2.55, 3.62) | 2.45 (2.13, 3.18) | 0.40 (0.35, 0.46) | 14.12** |

** *p* < 0.001; a: The β value associated between chronotype and lifestyle, b: OR value of lifestyle on mental health.

Adjusted for age, gender, country, education level, employment status, household income, and Townsend deprivation index.

**Table S10 Mediating role of specific lifestyle factors in the association between chronotype and depression**

| Mediating variable | Chronotype | a | b | Total effect | Direct effect | Indirect effect | Proportion, % |
| --- | --- | --- | --- | --- | --- | --- | --- |
| Smoking status | Definite morning chronotype | Ref | 1.51 (1.43, 1.59) |  |  |  |  |
|  | Intermediate chronotype | 1.23 (1.19, 1.27) |  | 0.40 (0.24, 0.52) | 0.36 (0.21, 0.48) | 0.04 (0.03, 0.05) | 9.23** |
|  | Definite evening chronotype | 2.31 (2.20, 2.43) |  | 2.23 (1.89, 2.52) | 2.03 (1.66, 2.31) | 0.21 (0.20, 0.23) | 9.40** |
| Alcohol intake | Definite morning chronotype | Ref | 1.01 (0.96, 1.06) |  |  |  |  |
|  | Intermediate chronotype | 1.12 (1.10, 1.15) |  | 0.40 (0.24, 0.51) | 0.40 (0.24, 0.52) | 0.00 (-0.00, 0.00) | 0.10 |
|  | Definite evening chronotype | 1.34 (1.29, 1.39) |  | 2.26 (1.91, 2.54) | 2.26 (1.91, 2.54) | 0.00 (0.00, 0.00) | 0.01 |
| Physical activity | Definite morning chronotype | Ref | 1.29 (1.24, 1.34) |  |  |  |  |
|  | Intermediate chronotype | 1.11 (1.09, 1.14) |  | 0.40 (0.25, 0.51) | 0.37 (0.22, 0.48) | 0.03 (0.02, 0.03) | 7.13** |
|  | Definite evening chronotype | 1.45 (1.40, 1.49) |  | 2.26 (1.91, 2.54) | 2.14 (1.79, 2.40) | 0.12 (0.10, 0.14) | 5.20** |
| Television viewing time | Definite morning chronotype | Ref | 1.19 (1.14, 1.24) |  |  |  |  |
|  | Intermediate chronotype | 1.10 (1.07, 1.12) |  | 0.40 (0.24, 0.51) | 0.38 (0.23, 0.50) | 0.01 (0.01, 0.02) | 3.68** |
|  | Definite evening chronotype | 1.32 (1.28, 1.37) |  | 2.26 (1.91, 2.54) | 2.21 (1.86, 2.48) | 0.05 (0.04, 0.06) | 2.26** |
| Sleep duration | Definite morning chronotype | Ref | 1.44 (1.38, 1.50) |  |  |  |  |
|  | Intermediate chronotype | 0.77 (0.75, 0.79) |  | 0.39 (0.24, 0.51) | 0.48 (0.32, 0.59) | -0.09 (-0.09, -0.08) | - |
|  | Definite evening chronotype | 1.11 (1.07, 1.15) |  | 2.26 (1.91, 2.54) | 2.22 (1.86, 2.48) | 0.04 (0.03, 0.06) | 1.89** |
| Fruit and vegetable intake | Definite morning chronotype | Ref | 1.19 (1.13, 1.25) |  |  |  |  |
|  | Intermediate chronotype | 1.19 (1.16, 1.22) |  | 0.39 (0.24, 0.51) | 0.38 (0.22, 0.49) | 0.02 (0.01, 0.02) | 3.93** |
|  | Definite evening chronotype | 1.61 (1.55, 1.68) |  | 2.26 (1.91, 2.53) | 2.20 (1.84, 2.47) | 0.06 (0.05, 0.07) | 2.66** |
| Oily fish intake | Definite morning chronotype | Ref | 1.07 (1.03, 1.11) |  |  |  |  |
|  | Intermediate chronotype | 1.08 (1.06, 1.10) |  | 0.40 (0.24, 0.51) | 0.39 (0.24, 0.51) | 0.01 (0.00, 0.01) | 1.42** |
|  | Definite evening chronotype | 1.18 (1.15, 1.22) |  | 2.26 (1.91, 2.54) | 2.25 (1.90, 2.54) | 0.01 (0.01, 0,02) | 0.66** |
| Red meat intake | Definite morning chronotype | Ref | 0.98 (0.93, 1.03) |  |  |  |  |
|  | Intermediate chronotype | 1.01 (0.99, 1.04) |  | 0.40 (0.24, 0.51) | 0.40 (0.24, 0.52) | 0. 00 (0.00, 0.00) | 0.01 |
|  | Definite evening chronotype | 1.17 (1.12, 1.22) |  | 2.26 (1.91, 2.54) | 2.26 (1.91, 2.54) | 0.00 (0.00, 0.00) | 0.01 |
| Processed meat intake | Definite morning chronotype | Ref | 1.08 (1.04, 1.13) |  |  |  |  |
|  | Intermediate chronotype | 1.21 (1.18, 1.23) |  | 0.40 (0.24, 0.51) | 0.38 (0.23, 0.50) | 0.01 (0.01, 0.01) | 3.09** |
|  | Definite evening chronotype | 1.40 (1.35, 1.45) |  | 2.26 (1,91, 2,54) | 2.23 (1.89, 2.51) | 0.03 (0.02, 0.03) | 1.20** |

** *p* < 0.001; a: The OR value of chronotype on lifestyle, b: The OR value of lifestyle on depression.

Adjusted for age, gender, country, education level, employment status, household income, and Townsend deprivation index.

**Table S11 Mediating role of specific lifestyle factors in the association between chronotype and anxiety**

| Mediating variable | Chronotype | a | b | Total effect | Direct effect | Indirect effect | Proportion, % |
| --- | --- | --- | --- | --- | --- | --- | --- |
| Smoking status | Definite morning chronotype | Ref | 1.36 (1.28, 1.45) |  |  |  |  |
|  | Definite evening chronotype | 2.31 (2.20, 2.43) |  | 1.38 (1.32, 1.56) | 1.25 (1.20, 1.40) | 0.13 (0.10, 0.16) | 9.10** |
| Alcohol intake | Definite morning chronotype | Ref | 1.02 (0.97, 1.07) |  |  |  |  |
|  | Definite evening chronotype | 1.34 (1.29, 1.39) |  | 1.39 (1.34, 1.58) | 1.39 (1.33, 1.58) | 0.00 (-0.01, 0.01) | 0.23 |
| Physical activity | Definite morning chronotype | Ref | 1.19 (1.14, 1.24) |  |  |  |  |
|  | Definite evening chronotype | 1.44 (1.40, 1.49) |  | 1.40 (1.34, 1.58) | 1.33 (1.27, 1.51) | 0.07 (0.07, 0.08) | 4.93** |
| Television viewing time | Definite morning chronotype | Ref | 1.15 (1.10, 1.20) |  |  |  |  |
|  | Definite evening chronotype | 1.32 (1.28, 1.37) |  | 1.40 (1.34, 1.58) | 1.36 (1.31, 1.54) | 0.04 (0.03, 0.05) | 2.52** |
| Sleep duration | Definite morning chronotype | Ref | 1.33 (1.27, 1.39) |  |  |  |  |
|  | Definite evening chronotype | 1.11 (1.07, 1.15) |  | 1.39 (1.33, 1.58) | 1.37 (1.31, 1.55) | 0.03 (0.02, 0.04) | 2.04** |
| Fruit and vegetable intake | Definite morning chronotype | Ref | 1.18 (1.12, 1.25) |  |  |  |  |
|  | Definite evening chronotype | 1.61 (1.55, 1.68) |  | 1.39 (1.33, 1.57) | 1.34 (1.29, 1.53) | 0.05 (0.04, 0.05) | 3.48** |
| Oily fish intake | Definite morning chronotype | Ref | 1.06 (1.01, 1.10) |  |  |  |  |
|  | Definite evening chronotype | 1.18 (1.15, 1.22) |  | 1.39 (1.34, 1.58) | 1.38 (1.32, 1.57) | 0.01 (0.01, 0.02) | 0.73** |
| Red meat intake | Definite morning chronotype | Ref | 0.91 (0.86, 0.97) |  |  |  |  |
|  | Definite evening chronotype | 1.17 (1,12, 1.22) |  | 1.39 (1.34, 1.58) | 1.39 (1.34, 1.58) | 0.00 (-0.00, 0.00) | 0.53 |
| Processed meat intake | Definite morning chronotype | Ref | 1.06 (1.02, 1.11) |  |  |  |  |
|  | Definite evening chronotype | 1.40 (1.35, 1.45) |  | 1.39 (1.34, 1.58) | 1.38 (1.33, 1.55) | 0.02 (0.01, 0.02) | 1.28** |

** *p* < 0.001; a: The OR value of chronotype on lifestyle, b: The OR value of lifestyle on anxiety.

Adjusted for age, gender, country, education level, employment status, household income, and Townsend deprivation index.

**Table S12 Sensitive analyses for association between lifestyle and incident depression and anxiety**

|  | **HR (95% CI)** | | |
| --- | --- | --- | --- |
|  | **Depression** | **Anxiety** |  |
| **Lifestyle score** |  |  |  |
| 0 (Most healthy) | Ref | Ref |  |
| 1 | 1.08 (1.00-1.16) | 1.11 (1.03-1.20) |  |
| 2 | 1.17 (1.09-1.26) | 1.13 (1.05-1.21) |  |
| 3 | 1.29 (1.20-1.39) | 1.19 (1.10-1.28) |  |
| 4 | 1.41 (1.31-1.53) | 1.22 (1.10-1.37) |  |
| 5 | 1.46 (1.32-1.61) | 1.26 (1.16-1.38) |  |
| ≥6 (Least healthy) | 1.65 (1.45-1.89) | 1.53 (1.32-1.77) |  |
| **Lifestyle category** |  |  |  |
| Most healthy | Ref | Ref |  |
| Moderately healthy | 1.22 (1.17-1.27) | 1.10 (1.06-1.15) |  |
| Least healthy | 1.48 (1.32-1.67) | 1.38 (1.21-1.58) |  |
| **Lifestyle factors** |  |  |  |
| Smoking status | 1.37 (1.30-1.44) | 1.15 (1.09-1.22) |  |
| Alcohol intake | 1.00 (0.95-1.05) | 1.00 (0.95-1.05) |  |
| Physical activity | 1.22 (1.17-1.26) | 1.11 (1.07-1.16) |  |
| Television viewing time | 1.16 (1.12-1.21) | 1.12 (1.08-1.17) |  |
| Fruit and vegetable intake | 1.17 (1.12-1.23) | 1.15 (1.09-1.21) |  |
| Oily fish intake | 1.07 (1.03-1.11) | 1.06 (1.02-1.11) |  |
| Red meat intake | 0.92 (0.87-1.03) | 0.85 (0.80-1.02) |  |
| Processed meat intake | 1.09 (1.05-1.14) | 1.07 (1.02-1.11) |  |

HR: hazard ratio; CI: confidence interval.

Adjusted for age, gender, country, education level, employment status, household income, Townsend deprivation index, and sleep duration.

**Table S13 Sensitive analyses for association of chronotype with incident depression and anxiety by lifestyle factors**

| **Lifestyle factors** | **HR (95% CI)** | |
| --- | --- | --- |
|  | **Depression** | **Anxiety** |
| **Smoking status (healthy)** |  |  |
| Definite morning chronotype | Ref | Ref |
| Intermediate chronotype | 1.11 (1.06-1.16) | 1.02 (0.97-1.07) |
| Definite evening chronotype | 1.51 (1.40-1.62) | 1.27 (1.17-1.38) |
| **Smoking status (unhealthy)** |  |  |
| Definite morning chronotype | Ref | Ref |
| Intermediate chronotype | 0.92 (0.83-1.03) | 0.88 (0.77-1.01) |
| Definite evening chronotype | 1.08 (0.93-1.24) | 1.14 (0.96-1.34) |
| **Alcohol intake (healthy)** |  |  |
| Definite morning chronotype | Ref | Ref |
| Intermediate chronotype | 1.10 (1.05-1.15) | 1.04 (0.94-1.15) |
| Definite evening chronotype | 1.47 (1.36-1.58) | 1.40 (1.21-1.61) |
| **Alcohol intake (unhealthy)** |  |  |
| Definite morning chronotype | Ref | Ref |
| Intermediate chronotype | 1.02 (0.97-1.07) | 0.94 (0.85-1.05) |
| Definite evening chronotype | 1.30 (1.20-1.41) | 1.18 (1.01-1.38) |
| **Physical activity (healthy)** |  |  |
| Definite morning chronotype | Ref | Ref |
| Intermediate chronotype | 1.09 (1.02-1.16) | 1.08 (1.02-1.14) |
| Definite evening chronotype | 1.46 (1.32-1.61) | 1.41 (1.29-1.53) |
| **Physical activity (unhealthy)** |  |  |
| Definite morning chronotype | Ref | Ref |
| Intermediate chronotype | 0.99 (0.93-1.06) | 1.01 (0.95-1.08) |
| Definite evening chronotype | 1.34 (1.20-1.49) | 1.20 (1.09-1.33) |
| **TV viewing time (healthy)** |  |  |
| Definite morning chronotype | Ref | Ref |
| Intermediate chronotype | 1.08 (1.02-1.14) | 1.09 (1.02-1.17) |
| Definite evening chronotype | 1.41 (1.30-1.53) | 1.48 (1.34-1.64) |
| **TV viewing time (unhealthy)** |  |  |
| Definite morning chronotype | Ref | Ref |
| Intermediate chronotype | 0.98 (0.93-1.04) | 1.03 (0.96-1.12) |
| Definite evening chronotype | 1.25 (1.14-1.36) | 1.28 (1.14-1.44) |
| **Fruit and vegetable intake (healthy)** |  |  |
| Definite morning chronotype | Ref | Ref |
| Intermediate chronotype | 1.10 (1.05-1.15) | 1.01 (0.91-1.12) |
| Definite evening chronotype | 1.49 (1.39-1.60) | 1.25 (1.09-1.44) |
| **Fruit and vegetable intake (unhealthy)** | |  |
| Definite morning chronotype | Ref | Ref |
| Intermediate chronotype | 1.00 (0.95-1.05) | 1.02 (0.91-1.14) |
| Definite evening chronotype | 1.23 (1.13-1.33) | 1.38 (1.18-1.60) |
| **Oily fish intake (healthy)** |  |  |
| Definite morning chronotype | Ref | Ref |
| Intermediate chronotype | 1.12 (1.06-1.18) | 1.05 (0.98-1.12) |
| Definite evening chronotype | 1.51 (1.38-1.65) | 1.39 (1.26-1.52) |
| **Oily fish intake (unhealthy)** |  |  |
| Definite morning chronotype | Ref | Ref |
| Intermediate chronotype | 1.00 (0.95-1.06) | 1.00 (0.93-1.08) |
| Definite evening chronotype | 1.24 (1.13-1.37) | 1.30 (1.17-1.44) |
| **Red meat intake (healthy)** |  |  |
| Definite morning chronotype | Ref | Ref |
| Intermediate chronotype | 1.10 (1.05-1.15) | 1.00 (0.89-1.12) |
| Definite evening chronotype | 1.50 (1.40-1.61) | 1.23 (1.04-1.46) |
| **Red meat intake (unhealthy)** |  |  |
| Definite morning chronotype | Ref | Ref |
| Intermediate chronotype | 1.02 (0.97-1.07) | 0.92 (0.81-1.04) |
| Definite evening chronotype | 1.32 (1.22-1.42) | 1.05 (0.86-1.27) |
| **Processed meat intake (healthy)** |  |  |
| Definite morning chronotype | Ref | Ref |
| Intermediate chronotype | 1.12 (1.06-1.18) | 1.01 (0.93-1.09) |
| Definite evening chronotype | 1.49 (1.38-1.62) | 1.35 (1.21-1.51) |
| **Processed meat intake (unhealthy)** |  |  |
| Definite morning chronotype | Ref | Ref |
| Intermediate chronotype | 1.03 (0.97-1.08) | 0.95 (0.87-1.03) |
| Definite evening chronotype | 1.31 (1.21-1.43) | 1.17 (1.03-1.33) |

HR: hazard ratio; CI: confidence interval.

Adjusted for age, gender, country, education level, employment status, household income, Townsend deprivation index and sleep duration.

**Table S14 Sensitive analyses of subgroup analyses for association of lifestyle with depression and anxiety**

|  | **HR (95% CI)** | |
| --- | --- | --- |
|  | **Depression** | **Anxiety** |
| **Female** |  |  |
| Most healthy | Ref | Ref |
| Moderately healthy | 1.26 (1.20-1.32) | 1.11 (1.06-1.17) |
| Least healthy | 1.62 (1.34-1.96) | 1.51 (1.22-1.87) |
| **Male** |  |  |
| Most healthy | Ref | Ref |
| Moderately healthy | 1.15 (1.08-1.22) | 1.08 (1.01-1.16) |
| Least healthy | 1.35 (1.16-1.58) | 1.29 (1.09-1.54) |
| **Age <60** |  |  |
| Most healthy | Ref | Ref |
| Moderately healthy | 1.30 (1.23-1.37) | 1.17 (1.11-1.24) |
| Least healthy | 1.51 (1.30-1.75) | 1.41 (1.19-1.67) |
| **Age ≥60** |  |  |
| Most healthy | Ref | Ref |
| Moderately healthy | 1.15 (1.08-1.21) | 1.05 (0.99-1.11) |
| Least healthy | 1.36 (1.11-1.67) | 1.25 (1.01-1.57) |

Adjusted for age, gender, country, education level, employment status, household income, Townsend deprivation index, and sleep duration.

**Table S15 Sensitive analyses for mediating role of lifestyle in the association of chronotype with depression and anxiety**

| **Outcome** | **Chronotype** | **a** | **b** | **Total effect** | **Direct effect** | **Indirect effect** | **Proportion, %** |
| --- | --- | --- | --- | --- | --- | --- | --- |
| Anxiety | Definite morning chronotype | Ref | 1.08 (1.06, 1.09) |  |  |  |  |
|  | Definite evening chronotype | 0.47 (0.45, 0.49) |  | 1.40 (1.32, 1.62) | 1.24 (1.17, 1.45) | 0.16 (0.14, 0.18) | 11.20** |
| Depression | Definite morning chronotype | Ref | 1.11 (1.09, 1.12) |  |  |  |  |
|  | Intermediate chronotype | 0.15 (0.14, 0.17) |  | 0.40 (0.35, 0.60) | 0.33 (0.29, 0.52) | 0.07 (0.07, 0.08) | 17.51** |
|  | Definite evening chronotype | 0.47 (0.45, 0.49) |  | 2.25 (2.01, 2.59) | 2.00 (1.76, 2.33) | 0.25 (0.22, 0.27) | 11.12** |

** *p* < 0.001; a: The β value associated between chronotype and lifestyle, b: OR value of lifestyle on mental health.

Adjusted for age, gender, country, education level, employment status, household income, Townsend deprivation index, and sleep duration.

**Table S16 Sensitive analyses for mediating role of specific lifestyle factors in the association between chronotype and depression**

| Mediating variable | Chronotype | a | b | Total effect | Direct effect | Indirect effect | Proportion, % |
| --- | --- | --- | --- | --- | --- | --- | --- |
| Smoking status | Definite morning chronotype | Ref | 1.51 (1.43, 1.59) |  |  |  |  |
|  | Intermediate chronotype | 1.24 (1.20, 1.28) |  | 0.40 (0.22, 0.54) | 0.36 (0.19, 0.51) | 0.04 (0.03, 0.04) | 9.23** |
|  | Definite evening chronotype | 2.32 (2.21, 2.44) |  | 2.24 (1.83, 2.74) | 2.03 (1.62, 2.51) | 0.21 (0.19, 0.23) | 9.44** |
| Alcohol intake | Definite morning chronotype | Ref | 1.01 (0.96, 1.06) |  |  |  |  |
|  | Intermediate chronotype | 1.12 (1.09, 1.15) |  | 0.40 (0.22, 0.55) | 0.40 (0.22, 0.55) | 0.00 (0.00, 0.00) | 0.11 |
|  | Definite evening chronotype | 1.34 (1.29, 1.39) |  | 2.26 (1.85, 2.76) | 2.26 (1.85, 2.76) | 0.00 (0.00, 0.00) | 0.06 |
| Physical activity | Definite morning chronotype | Ref | 1.29 (1.24, 1.34) |  |  |  |  |
|  | Intermediate chronotype | 1.11 (1.09, 1.13) |  | 0.40 (0.22, 0.55) | 0.37 (0.20, 0.52) | 0.03 (0.03, 0.03) | 7.66** |
|  | Definite evening chronotype | 1.45 (1.40, 1.49) |  | 2.27 (1.85, 2.77) | 2.15 (1.74, 2.64) | 0.12 (0.10, 0.13) | 5.30** |
| Television viewing time | Definite morning chronotype | Ref | 1.19 (1.14, 1.24) |  |  |  |  |
|  | Intermediate chronotype | 1.09 (1.07, 1.12) |  | 0.40 (0.22, 0.55) | 0.39 (0.21, 0.53) | 0.01 (0.01, 0.02) | 3.36** |
|  | Definite evening chronotype | 1.32 (1.27, 1.37) |  | 2.27 (1.86, 2.76) | 2.22 (1.81, 2.72) | 0.05 (0.04, 0.06) | 2.20** |
| Fruit and vegetable intake | Definite morning chronotype | Ref | 1.19 (1.13, 1.25) |  |  |  |  |
|  | Intermediate chronotype | 1.19 (1.16, 1.22) |  | 0.40 (0.22, 0.55) | 0.38 (0.21, 0.53) | 0.02 (0.01, 0.02) | 4.27** |
|  | Definite evening chronotype | 1.61 (1.55, 1.68) |  | 2.26 (1.85, 2.76) | 2.20 (1.80, 2.69) | 0.06 (0.05, 0.08) | 2.71** |
| Oily fish intake | Definite morning chronotype | Ref | 1.07 (1.03, 1.11) |  |  |  |  |
|  | Intermediate chronotype | 1.08 (1.06, 1.10) |  | 0.40 (0.22, 0.55) | 0.40 (0.22, 0.54) | 0.01 (0.00, 0.01) | 1.33** |
|  | Definite evening chronotype | 1.19 (1.15, 1.22) |  | 2.26 (1.85, 2.76) | 2.25 (1.84, 2.74) | 0.01 (0.01, 0.02) | 0.64** |
| Red meat intake | Definite morning chronotype | Ref | 0.98 (0.93, 1.04) |  |  |  |  |
|  | Intermediate chronotype | 1.01 (0.98, 1.03) |  | 0.40 (0.22, 0.55) | 0.40 (0.22, 0.55) | 0.00 (−0.00, 0.00) | - |
|  | Definite evening chronotype | 1.17 (1.12, 1.22) |  | 2.26 (1.85, 2.76) | 2.26 (1.86, 2.76) | 0.00 (−0.00, 0.00) | - |
| Processed meat intake | Definite morning chronotype | Ref | 1.08 (1.04, 1.13) |  |  |  |  |
|  | Intermediate chronotype | 1.21 (1.18, 1.23) |  | 0.40 (0.22, 0.55) | 0.39 (0.21, 0.54) | 0.01 (0.01, 0.02) | 3.15** |
|  | Definite evening chronotype | 1.40 (1.35, 1.45) |  | 2.26 (1.85, 2.76) | 2.24 (1.84, 2.74) | 0.03 (0.01, 0.04) | 1.22** |

** *p* < 0.001; a: The OR value of chronotype on lifestyle, b: The OR value of lifestyle on depression.

Adjusted for age, gender, country, education level, employment status, household income, Townsend deprivation index, and sleep duration.

**Table S17 Sensitive analyses for mediating role of specific lifestyle factors in the association between chronotype and anxiety**

| Mediating variable | Chronotype | a | b | Total effect | Direct effect | Indirect effect | Proportion, % |
| --- | --- | --- | --- | --- | --- | --- | --- |
| Smoking status | Definite morning chronotype | Ref | 1.36 (1.28, 1.44) |  |  |  |  |
|  | Definite evening chronotype | 2.32 (2.21, 2.44) |  | 1.39 (1.12, 1.77) | 1.26 (0.97, 1.61) | 0.13 (0.11, 0.16) | 9.04** |
| Alcohol intake | Definite morning chronotype | Ref | 1.02 (0.97, 1.07) |  |  |  |  |
|  | Definite evening chronotype | 1.34 (1.29, 1.39) |  | 1.40 (1.14, 1.79) | 1.40 (1.14, 1.78) | 0.00 (0.00, 0.01) | 0.24 |
| Physical activity | Definite morning chronotype | Ref | 1.19 (1.14, 1.24) |  |  |  |  |
|  | Definite evening chronotype | 1.45 (1.40, 1.49) |  | 1.40 (1.14, 1.79) | 1.33 (1.07, 1.71) | 0.07 (0.06, 0.08) | 4.98** |
| Television viewing time | Definite morning chronotype | Ref | 1.15 (1.10, 1.20) |  |  |  |  |
|  | Definite evening chronotype | 1.32 (1.27, 1.37) |  | 1.40 (1.14, 1.78) | 1.37 (1.11, 1.75) | 0.03 (0.02, 0.04) | 2.46** |
| Fruit and vegetable intake | Definite morning chronotype | Ref | 1.18 (1.12, 1.25) |  |  |  |  |
|  | Definite evening chronotype | 1.61 (1.55, 1.68) |  | 1.40 (1.13, 1.78) | 1.35 (1.09, 1.73) | 0.05 (0.04, 0.06) | 3.51** |
| Oily fish intake | Definite morning chronotype | Ref | 1.06 (1.01, 1.10) |  |  |  |  |
|  | Definite evening chronotype | 1.18 (1.15, 1.22) |  | 1.40 (1.14, 1.79) | 1.39 (1.13, 1.77) | 0.01 (0.01, 0.02) | 0.70** |
| Red meat intake | Definite morning chronotype | Ref | 0.91 (0.86, 0.99) |  |  |  |  |
|  | Definite evening chronotype | 1.17 (1.12, 1.22) |  | 1.40 (1.14, 1.78) | 1.41 (1.14, 1.79) | -0.01 (-0.01, -0.00) | - |
| Processed meat intake | Definite morning chronotype | Ref | 1.06 (1.02, 1.11) |  |  |  |  |
|  | Definite evening chronotype | 1.40 (1.35, 1.45) |  | 1.40 (1.14, 1.79) | 1.38 (1.13, 1.76) | 0.02 (0.00, 0.03) | 1.29** |

** *p* < 0.001; a: The OR value of chronotype on lifestyle, b: The OR value of lifestyle on anxiety.

Adjusted for age, gender, country, education level, employment status, household income, Townsend deprivation index, and sleep duration.

**Table S18 Characteristics of participants with (N=242,391) and without (N=118,146) lifestyle data**

| **Characteristics** | **With lifestyle (n=242,391)** | **Without lifestyle (n=118,146)** | ***P* values** |
| --- | --- | --- | --- |
| **Age, years (mean ± SD)** | 56.88 ± 8.01 | 56.99 ± 7.98 | <0.001 |
| **Gender** |  |  | <0.001 |
| Female | 130,401 (53.80) | 69,009 (58.41) |  |
| Male | 111,990 (46.20) | 49,137 (41.59) |  |
| **Country** |  |  | 0.0347 |
| England | 231,756 (95.61) | 112,780 (95.46) |  |
| Wales | 10,635 (4.39) | 5,366 (4.54) |  |
| **Education** |  |  | <0.001 |
| Higher | 75,660 (31.21) | 35,148 (29.75) |  |
| Upper secondary | 83,222 (34.33) | 37,716 (31.92) |  |
| Lower secondary | 13,329 (5.50) | 6,526 (5.52) |  |
| Vocational | 29,647 (12.23) | 13,328 (11.28) |  |
| No secondary education | 39,249 (16.19) | 23,940 (20.26) |  |
| Prefer not to answer | 1,284 (0.53) | 1,488 (1.26) |  |
| **Household income** |  |  | <0.001 |
| Less than £31,000 | 102,138 (42.14) | 47,619 (40.31) |  |
| £31,000 and above | 114,063 (47.06) | 48,637 (41.17) |  |
| Missing data | 26,190 (10.80) | 21,890 (18.53) |  |
| **Townsend deprivation index (mean ± SD)** | -1.65 ± 2.88 | -1.45 ± 2.99 | <0.001 |
| **Current employment status** |  |  | <0.001 |
| Employed | 138,706 (57.22) | 66,273 (56.09) |  |
| Retired | 85,807 (35.40) | 41,317 (34.97) |  |
| Unemployed, home | 16,521 (6.82) | 9,462 (8.01) |  |
| None of the above | 935 (0.39) | 653 (0.55) |  |
| Prefer not to answer | 422 (0.17) | 441 (0.37) |  |
| **Chronotype** |  |  | <0.001 |
| Definite morning chronotype | 66,255 (27.33) | 29,898 (25.31) |  |
| Intermediate chronotype | 156,206 (64.44) | 77,456 (65.56) |  |
| Definite evening chronotype | 19,930 (8.22) | 10,792 (9.13) |  |
| **BMI (mean ± SD)** | 27.38 ± 4.66 | 27.45 ± 4.90 | <0.001 |
| **Hypertension** | 66,332 (27.37) | 32,725 (27.70) |  |
| **Diabetes** | 11,724 (4.84) | 5,572 (4.72) |  |

Continues variables are displayed as means ± SD, and categorical variables are displayed as numbers (percentages).

Abbreviations: SD, standard deviation; BMI, body mass index.

**Table S19 ICD-10 codes used to ascertain anxiety and depression in the UK Biobank**

|  | **ICD-10 Code** |
| --- | --- |
| Depression | F32.0, F32.1, F32.2, F32.3, F32.8, F32.9, F33.0,  F33.1, F33.2, F33.3, F33.4, F33.8, F33.9 |
| Anxiety | F40.0, F40.1, F40.2, F40.8, F40.9, F41.0, F41.1, F41.2,  F41.3, F41.8, F41.9, F42.0, F42.1, F42.2, F42.8, F42.9,  F43.0, F43.1, F43.2, F43.8, F43.9 |

Abbreviations: ICD, International Classification of Diseases.

**Table S20** **Best-fitting parameters of the polygenetic risk scores for depression and anxiety**

| **Outcomes** | **AUC** | **Number of SNPs in GWAS summary statistics** |
| --- | --- | --- |
| Depression | 0.5592 | 9,874,287 |
| Anxiety | 0.5198 | 6,330,995 |

**Table S21 Variables used to create lifestyle score for UK Biobank**

| **Lifestyle factors** | **Questionnaire** | **‘Healthy’** | **‘Unhealthy’** |
| --- | --- | --- | --- |
| Smoking status | "Do you smoke tobacco now?" and "In the past, how often have you smoked tobacco?" | Past or never smoker | Current |
| Alcohol intake | “About how often do you drink alcohol?” | ≤ 4 times week | Daily or almost daily |
| Physical activity | IPAQ short form2– total time walking or moderate and vigorous-intensity physical activity (PA) in previous week | ≥150 min/week moderate or ≥ 75 min/week vigorous PA | <150 min/week moderate or < 75 min/week vigorous PA |
| TV viewing time | "In a typical day, how many hours do you spend watching TV?" | < 4 h/day | ≥ 4 h/day |
| Sleep time | “About how many hours sleep do you get in every 24 hours?” | >7 or <9h/day | <7 or >9h/day |
| Dietary characteristics | Individual dietary components contributed directly to lifestyle score. |  |  |
| Fruit and vegetable intake | "About how many of …. would you eat per day?” Separate questions for pieces of fresh and dried fruit, tablespoons of salad or cooked/raw vegetables. Combined and converted to g/day (1 portion = 80 g) | ≥ 400 g/day | <400 g/ day |
| Oily fish intake | "How often do you eat oily fish? (e.g. sardines, salmon, mackerel, herring)" | ≥1 portion/week | <1 portion/week |
| Red meat intake | "How often do you eat…?” Separate questions for Beef / lamb or mutton / pork (excluding processed meats such as ham or bacon). Red meat included due to clear link between red meat and mortality. | ≤3 portion/week | >3 portion/week |
| Processed meat intake | "How often do you eat processed meats (such as bacon, ham, sausages, meat pies, kebabs, burgers, chicken nuggets)?" | ≤1 portion/week | >1 portion/week |

Participants were classified to healthy or unhealthy categories based on their responses to questions for each lifestyle factor. UK Biobank physical activity (PA) data were analyzed in accordance with the International Physical Activity Questionnaire (IPAQ) scoring protocol with total physical activity computed as the sum of walking, moderate and vigorous activity, measured as metabolic equivalents (MET-hours/week). National dietary guidelines were used as the basis for the dietary components. UK Biobank dietary information was collected via the Oxford WebQ; a web-based 24-hour recall questionnaire developed specifically for use in large population studies.

**Table S22 Covariates definitions and assessment in our study**

| **Covariates** | **Description** | **Assessment** | **UK Biobank Code** |
| --- | --- | --- | --- |
| Age (year) | Age when attended assessment center | Based on date of birth and date of attending assessment centre | 21003 |
| Gender | Male, female | NHS had recorded for the participant and self-reported sex | 31 |
| Country | England, Wales | The UK Biobank assessment center at which participant consented | 54 |
| Education | Higher (College or University degree), Upper secondary (A levels/AS levels or equivalent, O levels/GCSEs or equivalent), Lower secondary (CSEs or equivalent), Vocational (NVQ or HND or HNC or equivalent, Other professional qualifications), No secondary education (None of the above) | Touchscreen question "Which of the following qualifications do you have? | 6138 |
| Household income | Less than £18,000, £ 18,000 to 30,999, £ 31,000 to 51,999, £ 52,000 to 100,000, Greater than 100,000 | Touchscreen question “What is the average total income before tax received by your HOUSEHOLD?” | 738 |
| Employment status | Current employment status | Touchscreen question “Which of the following describes your current situation?” | 6142 |
| Townsend deprivation index | Continuous | Townsend deprivation index calculated immediately prior to participant joining UK Biobank. | 189 |
| Diabetes | Yes, no | Touchscreen questionnaire and verbal interview: self-reported diabetes (diabetes, type 1 diabetes or type 2 diabetes) or insulin use | 2443, 6177, 20002 |
| Hypertension | Yes, no | Touchscreen questionnaire and verbal interview: self-reported hypertension or anti-hypertensive medication use | 6150, 6177, 20002 |
| Body mass index | Body mass index | Constructed from height and weight measured during the initial Assessment Centre visit. | 21001 |

**Figure S1 Sensitive analyses for the joint association of chronotype and genetic risk with depression and anxiety when excluding cases occurred in the first 2 years of follow-up (A) depression, (B) anxiety**

**
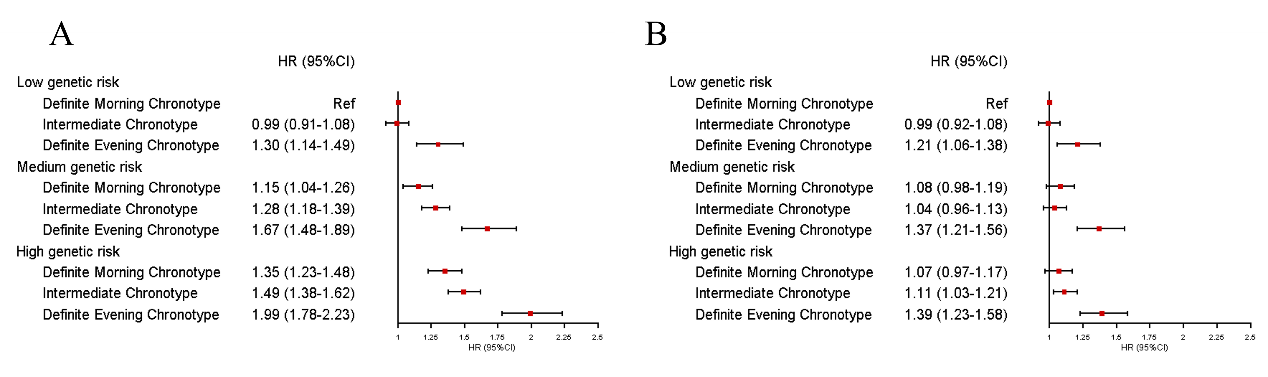
**

**Figure S2 Sensitive analyses for the joint association of chronotype and genetic risk with depression when excluding anxiety cases during follow-up**


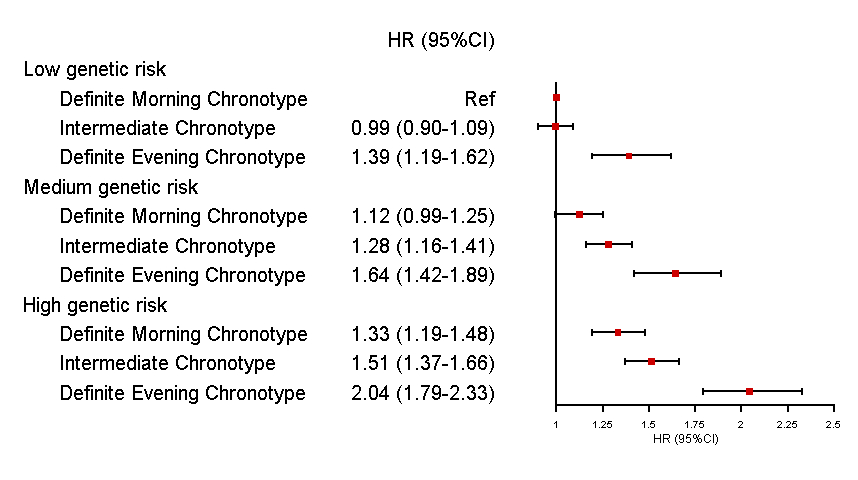


**Figure S3 Sensitive analyses for the joint association of chronotype and genetic risk with anxiety when excluding depression cases during follow-up**


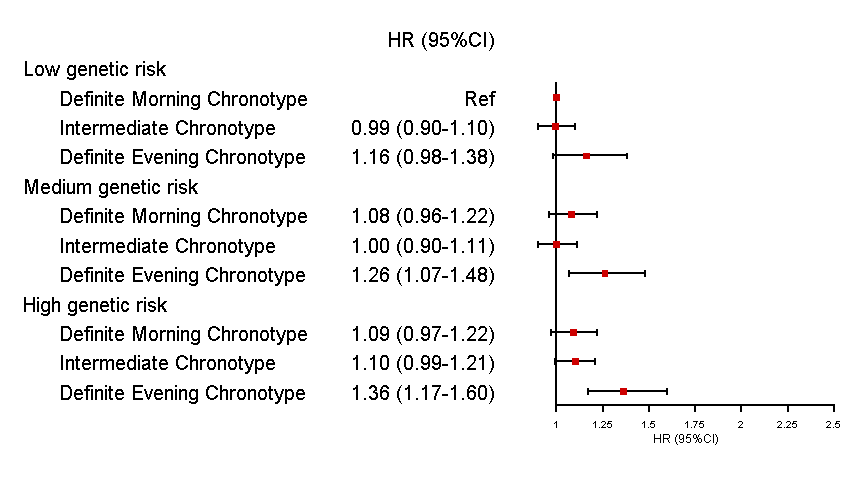


**Figure S4 Sensitive analyses for the joint association of chronotype and genetic risk with depression and anxiety when additional adjustment for other covariates including hypertension, diabetes, bmi, lifestyle score (A) depression, (B) anxiety**


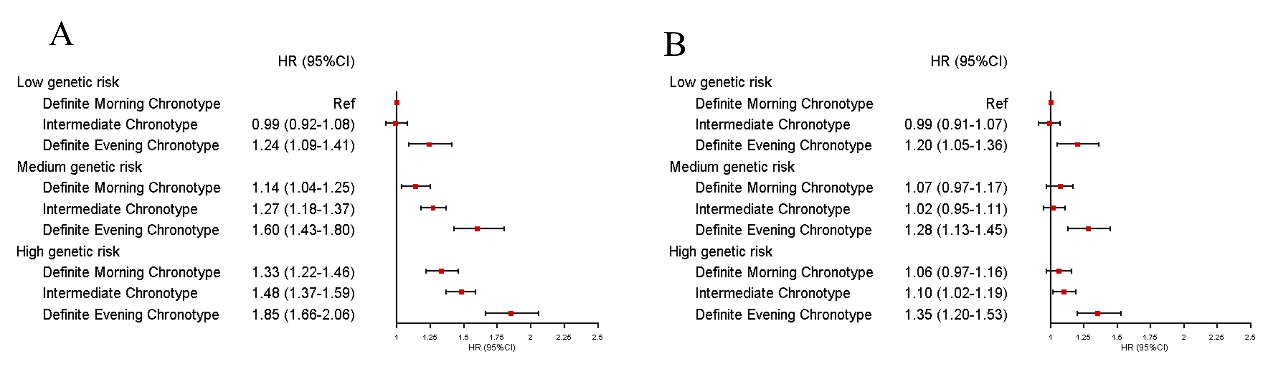


**Figure S5 Sensitive analyses for the joint association of chronotype and genetic risk with depression and anxiety when excluding missing covariates (A) depression, (B) anxiety**


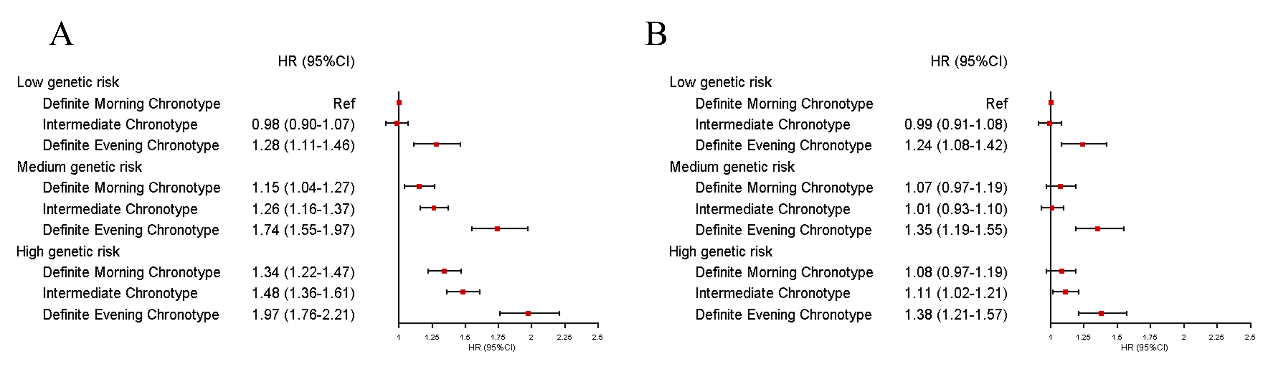


**Figure S6 Sensitive analyses for the joint association of chronotype and lifestyle with depression and anxiety when excluding cases occurred in the first 2 years of follow-up (A) depression, (B) anxiety**


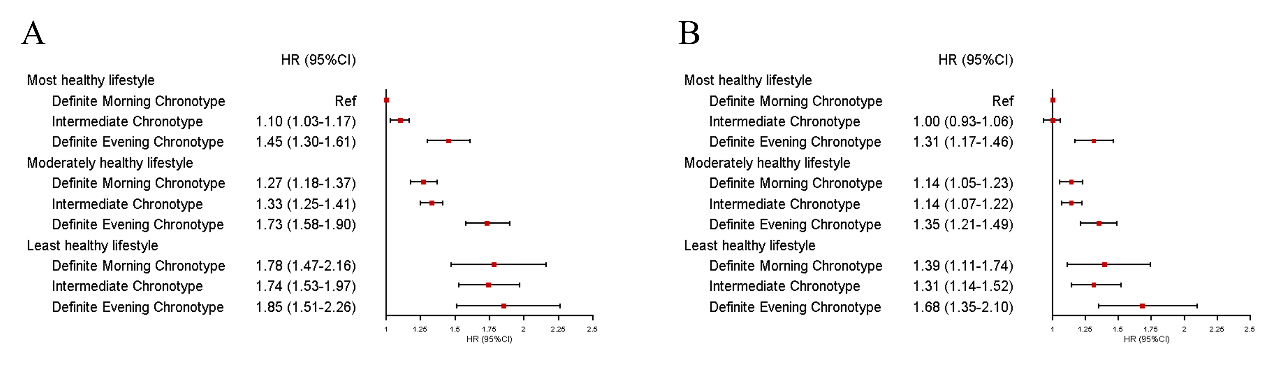


**Figure S7 Sensitive analyses for the joint association of chronotype and lifestyle with depression when excluding anxiety cases during follow-up**


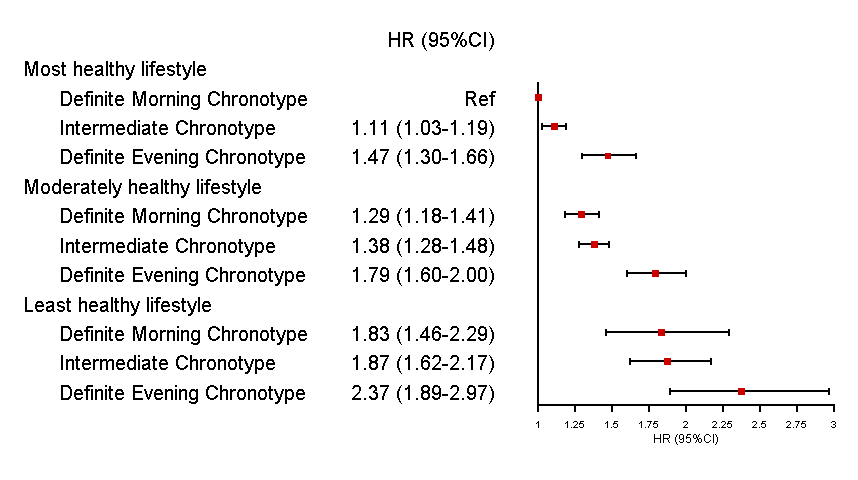


**Figure S8 Sensitive analyses for the joint association of chronotype and lifestyle with anxiety when excluding depression cases during follow-up**


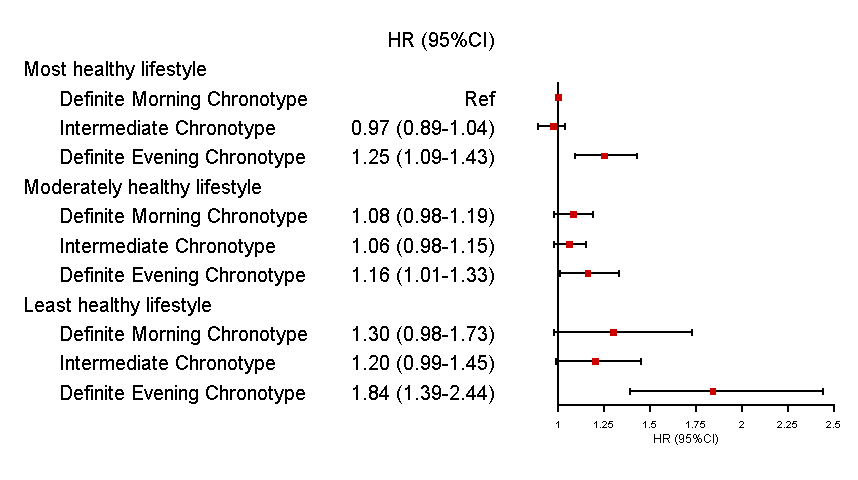


**Figure S9 Sensitive analyses for the joint association of chronotype and lifestyle with depression and anxiety when additional adjustment for other covariates including hypertension, diabetes, bmi, PRS (A) depression, (B) anxiety**


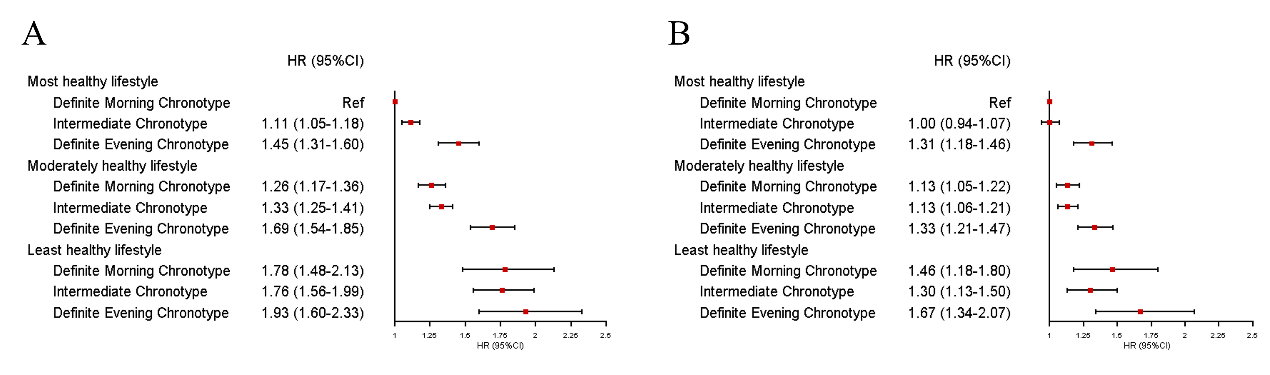


**Figure S10 Sensitive analyses for the joint association of chronotype and lifestyle with depression and anxiety when excluding missing covariates (A) depression, (B) anxiety**


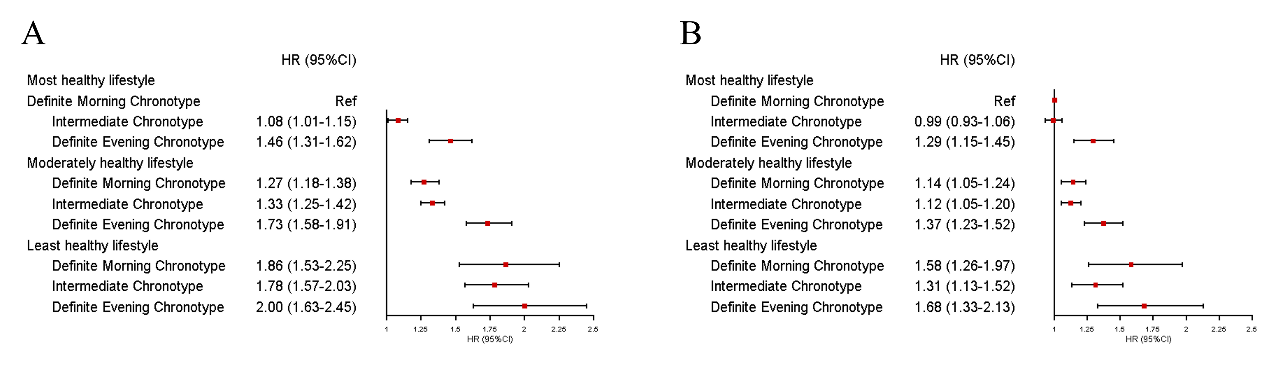


**Figure S11 Sensitive analyses for the joint association of chronotype and lifestyle with depression when sleep duration was not included in lifestyle**


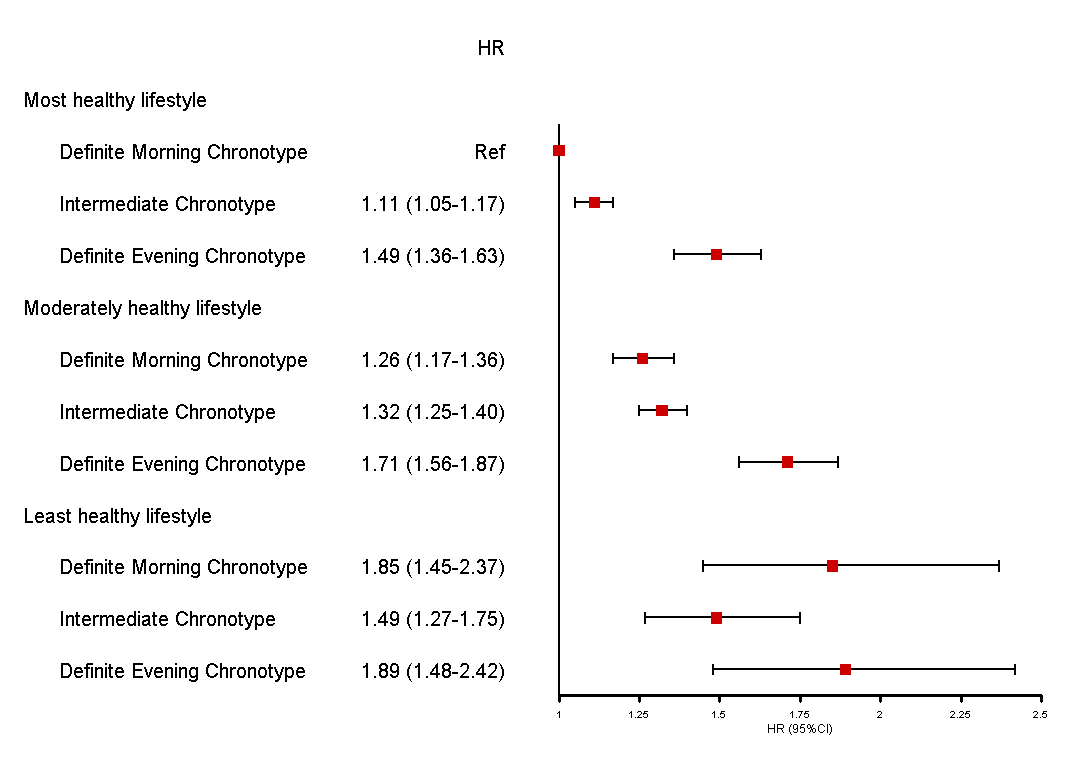


**Figure S12 Sensitive analyses for the joint association of chronotype and lifestyle with anxiety when sleep duration was not included in lifestyle**


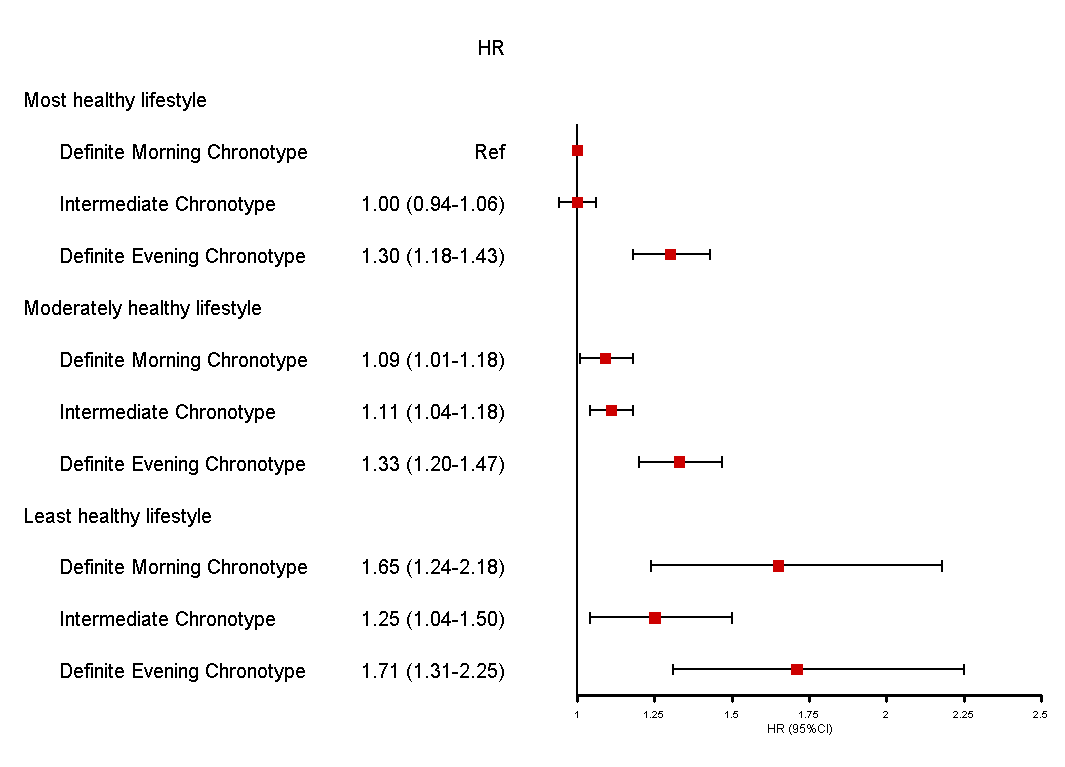


**Figure S13** **Flowchart of the study**

496,022 participants

Excluded: Participants with depression or anxiety at baseline (N=6,379)

UK Biobank (N=502,401)

360,537 participants

438,512 participants

Excluded: Participants without chronotype at baseline (N=58,312)

Excluded: Participants with missing information on genetic data of depression or anxiety (N=77,975)

242,391 participants for the association of chronotype, genetic risk, lifestyle with anxiety and depression

Excluded: Participants with missing information on lifestyle (N=118,146)

**Figure S14 Directed Acyclic Graph for the association of chronotype with depression and anxiety**

**
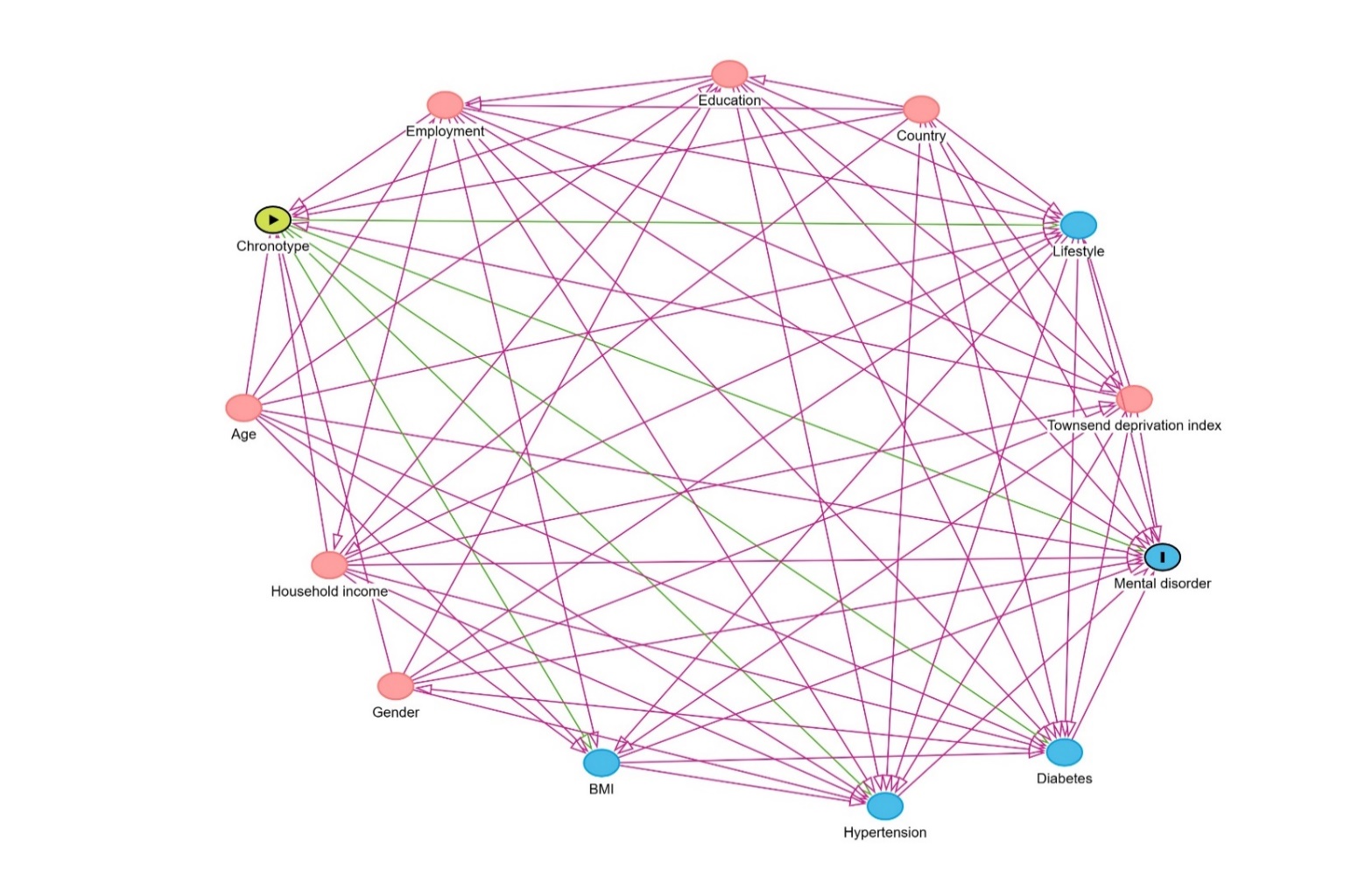
**
